# Repetitive Transcranial Magnetic Stimulation for Neuropathic Pain on the Non-Motor Cortex: An Evidence Mapping of Systematic Reviews

**DOI:** 10.1155/2021/3671800

**Published:** 2021-10-29

**Authors:** Yaning Zang, Yongni Zhang, Xigui Lai, Yujie Yang, Jiabao Guo, Shanshan Gu, Yi Zhu

**Affiliations:** ^1^Department of Kinesiology, Shanghai University of Sport, Shanghai, China; ^2^School of Health Sciences, Duquesne University, Pittsburgh, PA, USA; ^3^Department of Biomedical Sciences, City University of Hong Kong, Kowloon, Hong Kong, China; ^4^Department of Rehabilitation Medicine, The Second School of Clinical Medicine, Xuzhou Medical University, Xuzhou, Jiangsu, China; ^5^Department of Physical Therapy, University of Toronto, Toronto, ON, Canada; ^6^Department of Musculoskeletal Pain Rehabilitation, The Fifth Affiliated Hospital of Zhengzhou University, Zhengzhou, Henan, China

## Abstract

**Objective:**

This study was aimed to summarize and analyze the quality of the available evidence in systematic reviews (SRs) of repetitive transcranial magnetic stimulation (rTMS) on the non-motor cortex (non-M1) for neuropathic pain (NP) through an evidence mapping approach.

**Methods:**

We follow the Global Evidence Mapping (GEM) methodology. Searches were conducted in PubMed, EMBASE, Epistemonikos, and the Cochrane Library. The study type was restricted to SRs with or without meta-analysis. All literature published before January 23, 2021, were included. The methodological quality of the included SRs was assessed using A Measurement Tool to Assess Systematic Reviews (AMSTAR-2). Data were extracted according to a defined population-intervention-comparison-outcome (PICO) framework from primary studies that included SRs. The same PICO was categorized into PICOs according to interventions (stimulation target, frequency, number of sessions (short: 1–5 sessions, medium: 5–10 sessions, and long: >10 sessions)) and comparison (sham rTMS or other targets). The evidence mapping was presented in tables and a bubble plot.

**Results:**

A total of 23 SRs were included. According to the AMSTAR-2, 20 SRs scored “very low” in terms of methodological quality, 2 SRs scored “low,” and 1 SR scored “high.” A total of 17 PICOs were extracted. The dorsolateral prefrontal cortex (DLPFC) is the most studied of the non-motor cortex targets. PICOs of DLPFC, premotor cortex (PMC), frontal cortex, and secondary somatosensory cortex (S2) were mainly categorized with a “potentially better” conclusion. High-frequency (5–20 Hz) rTMS of non-M1 usually lead to “potentially better” conclusions.

**Conclusions:**

DLPFC, PMC, frontal cortex, and S2 seem to be promising new targets for rTMS treatment of certain NP. Evidence mapping is a useful and reliable methodology to identify and present the existing evidence gap that more research efforts are necessary in order to highlight the optimal stimulation protocols for non-M1 targets and standardize parameters to fill the evidence gaps of rTMS. Further investigation is advised to improve the methodological quality and the reporting process of SRs.

## 1. Introduction

Neuropathic pain (NP) is a chronic pain caused by lesions or dysfunction of the peripheral or central nervous system; it is often characterized by persistent pain, hyperalgesia, or even spontaneous pain [1]. NP not only disturbs daily activities, work, and sleep but also increases the incidence of emotional disorders such as patient depression and anxiety [2]. The mechanisms of NP are still unclear, which lead to the challenge of NP prevention and management. Pathological changes such as spontaneous activity in damaged non-nociceptive fibers, peripheral and central, hyperactivity in nociceptors, and changes in central neuroplastic may be the possible reasons for NP [3, 4]. Currently, pharmacological treatment is the primary treatment for NP, including tricyclic anti-depressants, anti-convulsants, anti-epileptics, non-steroidal anti-inflammatory drugs, opioids, and so on. [5, 6]. However, pharmacological treatments provide less satisfaction with pain relief in many patients. In addition, drugs cause many adverse effects and even lead to drug dependence and abuse [7], wherein recommendation levels are not high [2, 8]. Thus, the treatment of NP remains a major unmet need, and the exploration of alternative approaches, especially evidence-based non-pharmacological interventions, is particularly important.

Repetitive transcranial magnetic stimulation (rTMS), as a non-invasive, safe non-pharmacological treatment, has been widely applied for NP. The rTMS technique uses magnetic pulses from an external stimulator to target specific cortical areas to generate induced currents that can alter the action potential of cortical nerve cells, induce depolarization of neurons, and ultimately lead to functional and even structural plasticity changes in the nervous system [9]. rTMS for NP has been published extensively. Stimulation target, frequency, and session are considered to be critical variables for analgesic efficacy. In terms of target, primary motor cortex (M1) is a commonly used stimulation target for rTMS and has been used for pain relief related to poststroke central pain, postherpetic neuralgia, and trigeminal facial pain. Although M1 has shown some efficacy in the treatment of NP, some studies have found that patients do not respond to M1 stimulation or only have short-lived effects. One study found the overall effectiveness rate was only about 40% [10]. This leaves a large gap in the search to find better management options for non-responders. Given the complexity of the disease type and the unclear mechanism of NP, M1 is not the suitable stimulation target for all types of NP. Therefore, the lack of individualized targeted therapy based on the characteristics of plasticity [11] may explain why rTMS is effective only in some patients, with a non-persistent efficacy and pain recurrence.

However, evidence-based evidence on non-M1 stimulation targets, treatment parameters, and treatment efficacy for the treatment of NP is still to be provided. Traditionally, SRs are a common methodology for evidence synthesis. SRs tend to focus on specific types of pain, whereas compared with M1, studies in non-M1 targets are insufficient and unfocused on specific NP, deeming SRs unable to provide a comprehensive overview of non-M1 regions for the treatment of NP. To overcome this barrier, an emerging synthesis method, evidence mapping [12–14], has been developed to provide an overview of the research area. Evidence mapping can provide both the breadth of evidence by extracting and analyzing primary data in SRs and the credibility of evidence by AMSTAR-2. The knowledge gaps identified by using evidence mapping can also inform future studies. This study aims to summarize, identify, and analyze the currently available evidence in SRs regarding rTMS on non-M1 for NP. This information is provided in a user-friendly manner that helps identify research gaps and assist evidence users in decision-making.

## 2. Methods

### 2.1. Setting the Boundaries and Context of the Evidence Mapping

**Figure 1:**
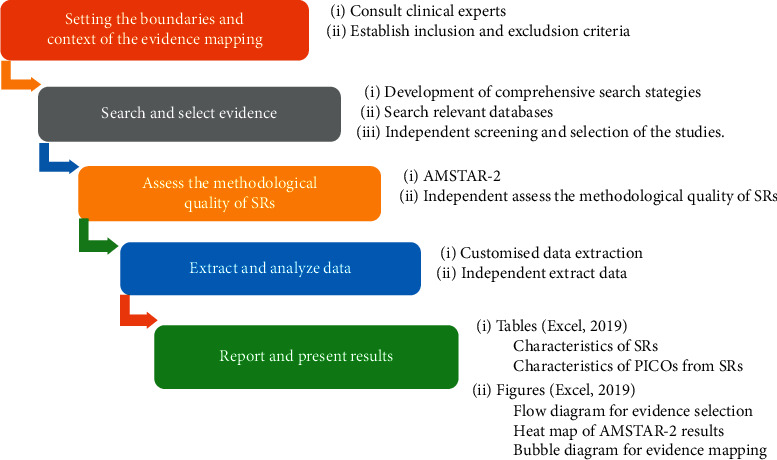
Core tasks for conducting evidence mapping.

This evidence mapping is based on the methodology proposed by GEM [15] and previous key studies [16–18]. The study process was divided into five stages (Figure 1). Studies and guidelines related to NP were referred, and an expert with a research background in NP was consulted to frame the evidence mapping. With the help of experts in this area, the specific terminology of the search strategy was confirmed, and the possible evidence users (pain, neurology, psychiatry, anesthesiology, and rehabilitation) involved were discussed. On the basis of the above information, the eligibility criteria have been established for inclusion in the study. Studies containing rTMS for NP were considered eligible. Studies on patients with NP were included, whereas experimental subjects that were animals or healthy people were excluded. The intervention should be rTMS, and the comparison could be rTMS, sham rTMS, other treatments of relieving pain, or no treatment. The outcome should be pain measured with various clinically validated tools such as visual analog scale (VAS), numerical rating scale (NRS), short-form McGill Pain Questionnaire, and brief pain inventory. Studies that did not address intervention outcomes, such as those aimed to explore NP-related pathophysiology and focusing on cost-effectiveness, were excluded. Studies that reported other outcomes (e.g., fatigue, motor function, spasticity, sensory function, and cognition) with the exception of pain were also excluded. Only SRs (with or without meta-analysis) were included as they could provide more reliable evidence. Literature published in non-English languages were excluded. Posters and conference abstracts were excluded.

### 2.2. Search and Select Evidence

We conducted searches of systematic literature published before January 23, 2021, on PubMed, EMBASE, Epistemonikos, and the Cochrane Library. Medical subject headings were used in combination with free-text terms for the search, such as “neuralgia,” “neurodynia,” “atypical neuralgia,” “nerve pain,” and “stump neuralgia.” Literature published in non-English languages were excluded. In addition, references of included studies were also searched to ensure the integrity of the search. The details of the search strategies are provided in Supplementary Material 1.

EndNote (version X9) was used to manage all retrieved results. After removing duplicated SRs, two reviewers (Zang and Lai) independently screened titles and abstracts to exclude irrelevant studies. Full-text studies were obtained and reviewed to make a terminal decision. Any disagreements in the decision-making process were resolved by negotiation or discussion with a third reviewer (Zhang).

### 2.3. Assessing the Methodological Quality of SRs

**Figure 2:**
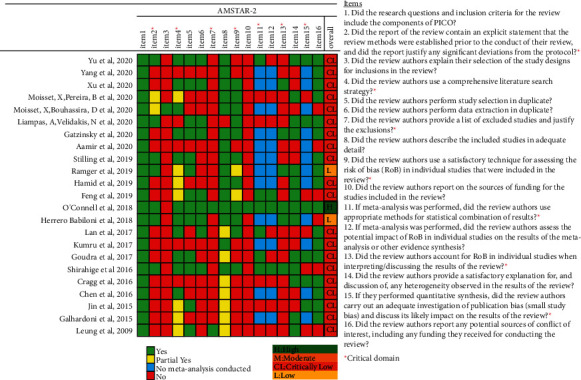
Methodological quality of the included systematic reviews.

The methodological quality for each SRs was assessed with the AMSTAR-2 [19]. Almost half of published SRs have included both randomized controlled trials (RCT) and non-randomized studies. AMSTAR-2 is suitable for evaluating the methodological quality of SRs that include RCT and non-RCT. A total of 16 items were included, covering the entire process of SRs and including topic selection, design, registration, data extraction, data statistical analysis, and discussion. AMSTAR-2 recommends 7 items (items: 2, 4, 7, 9, 11, 13, and 15) as key items for evaluating the quality of SRs (Figure 2). According to the absence of items, the evaluation results of the SRs are divided into the following four categories: “high,” no key items missing and on more than one non-critical item missing; “moderate,” no key items missing and more than one non-key item missing; “low,” one key item missing and with or without non-key items missing; and “critically low,” more than one key item missing and with or without non-critical items missing.

### 2.4. Extract and Analyze Data

Two data extraction tables were designed to record the main characteristics of the included SRs. Data were grouped into two categories:

- General characteristics of the SRs: authors, years of publication, types of SRs (with or without meta-analysis), objectives, dates of search, sample sizes, designs, and numbers of included primary studies.
- Characteristics of research questions: the PICO framework was used to extract data from primary studies that had been included in SRs. The four key components are study population, interventions, comparative measures, and assessment methods for outcomes. Due to the unavoidable heterogeneity of rTMS protocol among studies, it is difficult to classify and categorize all parameters. Targets, frequency, and sessions were most often reported for each primary study included in the SRs. They have been shown to influence analgesic effects and are identified as the most clinically significant factors [20–23]. High and low frequencies of rTMS could induce transient excitatory and inhibitory effects, respectively [24]. Sessions of rTMS are considered to be an important factor in maintaining the effects. Thus, the PICO characteristics are mainly focused on interventions (targets, frequency, and sessions) and comparison (sham rTMS or other targets).

According to the criteria reported previously, the conclusions of rTMS on NP reported by the systematic evaluation were classified into five categories: “potentially better,” “mixed results,” “unclear,” “no difference,” and “potentially worse.” “Potentially better” is defined as statistically significant efficacy of rTMS, with the authors of the SR having no doubt about the current evidence and recommending the therapy. “Mixed results” means that the results of SRs with similar content are controversial (e.g., some SRs found no difference between transcranial magnetic stimulation with the control group in the same study, whereas others found potential benefits of transcranial magnetic stimulation over the control group. “Unclear” is defined as the SR authors concluding that the evidence is inconclusive or that the conclusions of a specific study were not reported by the authors of the SR. “No difference” is defined as comparable efficacy of rTMS as compared to the control group or no statistical difference. “Potentially worse” is defined as better efficacy in the control group as compared with rTMS. When SRs yielded consistent results for the same study, it was added to the appropriate group, and conflicting results were included in the “mixed results” group.

Two authors (Zang and Lai) assessed the methodological quality and extracted data independently. Any difference of opinions was discussed with the third author (Zhang). The original authors were contacted for missing information when necessary.

### 2.5. Report and Present Results

The evidence mapping was presented in three visualizations, and the findings were summarized in a narrative synthesis:

- Tables were used to describe the basic characteristics of the included SRs and characteristics of all identified PICOs.
- A heat map was displayed to present the quality of SRs.
- A bubble plot was used to present a comprehensive visualization of the conclusions of included SRs, methodological quality, sample size, and distribution of interventions. The bubble plot can display the following information: (1) authors' conclusions: ratings on the *x*-axis are: “potentially better,” “mixed results,” “unclear,” “no difference,” or “potentially worse”; (2) AMSTAR-2 evaluation results: presented in four different colors on the *y*-axis (red indicating critically very low, orange indicating low, yellow indicating medium, and green indicating high quality); (3) research characteristics: different colored bubbles indicate different PICOs; (4) the number of primary studies included in the SRs, shown in each bubble and indicated by the bubble size; and (5) interpretation of bubble plot: some primary studies may be included in multiple SRs. If SRs synthesized different conclusions for the same primary study, the same PICOs would appear in different classifications on the *X*-axis. If the same primary study was included by SRs of different quality, then the same PICOs classified by the primary study would appear in different classifications on the *Y*-axis. The included SRs covering similar topics may have overlapped considerably in terms of the primary studies they contained. Therefore, when interpreting the evidence mapping, it is critical that all figures in the bubble are not added up and that any overlapping studies are removed. Due to SRs serving as the unit of analysis rather than the primary study, the risk of bias is reduced when multiple reviews reach the same conclusion. When higher-quality SRs cover the same primary study, these findings may be interpreted with more confidence than the findings of lower quality reviews [25]. Conversely, the potential for bias appears when the primary study is concluded by only one low-quality SR, presented as “critically low” at the bottom of the bubble plot. Multiple bubbles with different results may indicate that this type of evidence highlights the preliminary stage or unclear nature of the evidence.

## 3. Results

### 3.1. Studies Selected

**Figure 3:**
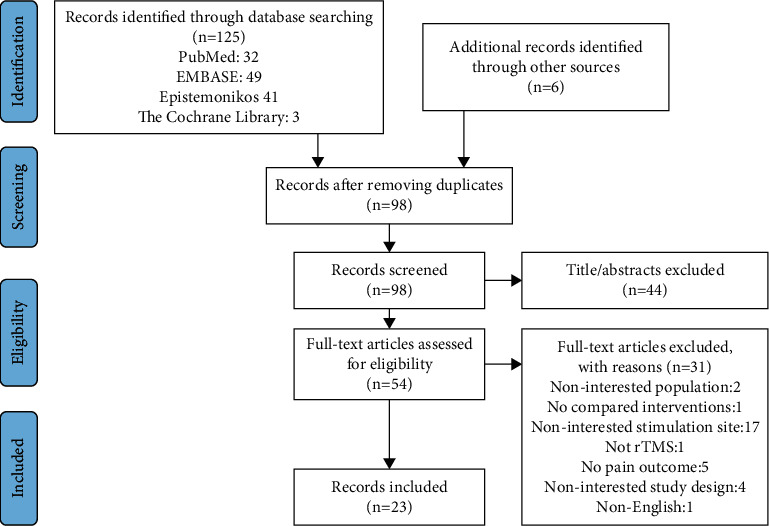
Flow diagram for evidence selection.

The study selection process is shown in Figure 3. The list of excluded studies and the reasons for exclusion are provided in Supplementary Material 2.

### 3.2. The Methodological Quality of SRs

As shown in Figure 2, according to AMSTAR-2 criteria, 1 Cochrane SR [23] was graded as “high.” Two SRs [26, 27] were graded as “low,” and 20 SRs [18, 27–45] were graded as “critically low” [22, 28–46]. The SRs were downgraded mainly due to the following reasons: absence of a predesigned and registered protocol [22, 29, 30, 34, 36, 40, 42–46]; no explanation for the selection of study design included in the SRs [22, 23, 26, 31, 33–46]; no list of the excluded studies or reasons for the exclusion [22, 26–45]; no statement of the funding or support for each included primary study in the SRs [22, 26–46]; and no investigation of the impact of the risk of bias in the included studies on the overall effect [28, 29, 31, 40, 42–46]. The detailed evaluation process is provided in Supplementary Material 3.

### 3.3. Characteristics of SRs

Table 1 shows the characteristics of the included SRs. All SRs [22, 23, 26–46] were published between 2009 and 2020. Among the 23 included SRs, 11 [23, 28, 31, 33, 37, 38, 40, 42, 44, 46] conducted a meta-analysis. The number of included primary studies ranged from 5 to 131, and they were conducted between 2004 and 2020. Each SR included patients ranging from 109 to 15,776. A total of 3 SRs [33, 39, 43] did not report or incompletely reported the designs of the included studies. Among the available data, a total of 509 randomized controlled trials (RCTs) accounted for 80.3% of the included studies in all SRs. Of all SRs, 13 [23, 27, 28, 30, 32, 36, 38, 41, 42, 45, 46] included only RCTs; 12 SRs [22, 23, 29, 31, 34, 36, 39, 40, 42, 44–46] included patients with NP with different causes; and 11 were specially conducted on NP with specific etiologies or due to a single disease. One SR [28] included pain after spinal cord injury (SCI); 4 SRs [26, 30, 33, 43] included central poststroke pain after stroke (CPSP); 4 SRs [31, 37, 38, 41] included migraine, 1 SR [35] included headache, and 1 SR [27] included orofacial pain (OFP). As for the intervention, 9 SRs [22, 29, 34, 36, 38, 40, 44–46] only assessed TMS; 8 SRs [23, 26, 28, 35, 37, 39, 41] also assessed other non-invasive stimulations; 1 SR [31] assessed neuromodulation techniques; 1 SR [30] assessed non-pharmacological interventions; 3 SRs [32, 33, 42] assessed pharmacological and non-pharmacological management of NP; and 1 SR [43] assessed non-invasive physical modalities.

### 3.4. Characteristics of PICOs from SRs

After merging the duplicated primary studies included in the 23 SRs, 24 primary studies that provide the mandatory parameter information were integrated into 17 PICOs groups according to the PICO characteristics.

The key characteristics of PICOs are listed in Table 2. The details of the characteristics are enumerated in Supplementary Material 4. In terms of the stimulation target, 6 PICOs stimulated the left DLPFC; 2 PICOs stimulated the S2; 2 PICOs stimulated the vertex; 1 PICO stimulated the PFC; 1 PICO stimulated the frontal cortex; 4 PICOs stimulated multiple different targets; and 1 PICO stimulated over the superior trapezius muscle. In terms of the stimulation frequency, 14 PICOs used high-frequency rTMS (>1 Hz); 2 PICOs used low-frequency rTMS (<1 Hz); and 1 PICO used both high and low frequencies. In terms of the number of sessions, 1–5 sessions were considered as short sessions, 6–10 as medium sessions, and more than 10 as long sessions. Three PICOs had long sessions; 6 PICOs had medium sessions; and 8 had short sessions. All PICOs used sham stimulation or placebo as a control to study the effectiveness of rTMS in patients with NP. In addition, 3 PICOs also compared the efficacy of different targets. The PICOs were concentrated in the following characteristics: 10 Hz, short-term sessions (4 PICOs from 7 primary studies); 10 Hz, medium-term sessions (3 PICOs from 6 primary studies); and 10 Hz, long-term sessions (2 PICOs from 5 primary studies).

### 3.5. Specific Findings from SRs in the Evidence Mapping

**Figure 5:**
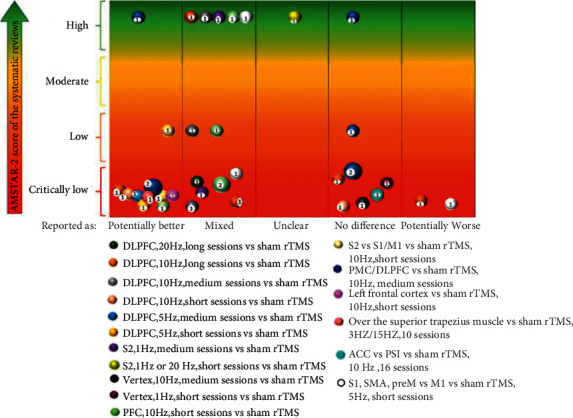
Evidence mapping of the rTMS on NP. Short: 1–5 sessions, medium: 5–10 sessions, and long: >10 sessions. rTMS = repetitive transcranial magnetic stimulation; DLPFC: dorsolateral prefrontal cortex; S2: secondary somatosensory cortex; PFC: prefrontal cortex; PMC: premotor cortex; ACC: anterior cingulate cortex; PSI: posterior superior insula; S1: postcentral gyrus; SMA: supplementary motor area; and preM: premotor area.

The evidence mapping of the rTMS for NP is presented in Figure 5. The bubble diagram is a visual display of data represented in Table 2. Evidence mapping showed that DLPFC is the most studied of the targets (5 PICOs from 9 primary studies) and showing a majority of “potentially better” treatment effects. In addition, the PICOs of PMC, frontal cortex, and S2 in trigeminal NP were mainly categorized with a “potentially better” conclusion and seem to be promising new targets for rTMS treatment of certain NP. PFC, S1, SMA, preM, and S2 in chronic visceral pain were categorized as “mixed” conclusions.

High-frequency (5–20 Hz) rTMS of non-M1 usually lead to “potentially better” conclusions as compared with sham stimulation, although some had transient effects. In contrast, the synthesis results for the lower frequencies (1 Hz) showed either no difference, unclear, or mixed.

Nine PICOs included 10 primary studies rated as “potentially better,” and one of these PICOs involved one primary study that was also included in a high-quality meta-analysis [47]. In accordance with the AMSTAR-2 quality assessment, the interventions in these four PICOs were considered beneficial in most cases. Six PICOs included 7 primary studies with different findings within different SRs that were rated as “mixed,” and this implies that the interventions in these eight PICOs had limited confidence in the effect estimates; true effects may be different from the study reports [48]. One PICO conclusion was rated as “unclear” due to its effect and was not reported in the SR [23]. Six PICOs included 7 primary studies that concluded that rTMS showed no difference when compared with the controls. Of these, 4 PICOs included 5 primary studies showing a potentially better effect of rTMS in the short term but no difference during long-term follow-up (Table 2; Supplementary Material 4). After the exclusion of studies that were ineffective during follow-up, no primary studies were included by a high-quality meta-analysis [47]. Two PICOs included 2 primary studies that showed a “potentially worse” conclusion. This finding indicated less effectiveness of these intervention protocols or inapplicability to particular NP, and the treatment effects could be uncertain [49].

## 4. Discussion

As far as we know, this evidence mapping may be the first synthesis of evidence on non-M1 targets for the treatment of NP. Following the classification criteria for interventions, this evidence mapping has described and organized existing evidence for non-M1 targets for NP. The majority of non-M1 targets reported as “potentially better” were DLPFC, probably due to DLPFC can coordinate the interaction between the cognitive pathway and the “pain matrix”, or play a direct role in promoting or inhibiting pain through the nociceptive downstream inhibitory pathway [25, 47]. In addition, the PMC, the left frontal cortex, and the S2 also seem to be promising therapeutic targets. These targets are also importantly involved in nociceptive modulation; they share some common mechanisms, such as the involvement in altering human temperature pain thresholds [50], inducing striatal dopamine release that modulates pain [51], and causing cerebral hemodynamic changes in broader cortical regions (e.g., cingulate cortex, anterior frontal cortex, thalamus, and other subcortical areas involved in pain modulation) [52]. Another important finding was that most studies tend to suggest that high-frequencies produce better effects [49, 53–61]. This may be due to the factor that high-frequency rTMS can directly excite the injured hemisphere, thus directly improving pain. However, this evidence mapping does not significantly find the specific number of sessions that may possibly lead to a “potentially better” conclusion. This may be due to the small number of primary studies of different PICOs, making the differences insignificant.

We were able to identify some research gaps by this evidence mapping to orient further research. (1) The included SRs have covered most non-M1 targets of NP, including the DLPFC, ACC, PSI, S2, and S1, while there are still evidence gaps for other targets, such as the premotor cortex (PMC), the supplementary motor complex (SMC), and so on. (2) Low-frequency rTMS inhibited the non-injured side and the high-frequency rTMS excited the injured side. Evidence needs to be evaluated as to whether low and high frequency are used simultaneously to achieve a rebalancing of reciprocal inhibition in both hemispheres and whether the therapeutic effect can be enhanced. (3) Studies have only been conducted from a single cortical target to a deep area of the brain; however, the fact is that any deep area is functionally connected to multiple superficial cortices. It would be interesting to examine whether combining different stimulation targets for treatment would enhance the analgesic effect. Similarly, combining transcranial direct current stimulation (tDCS) or prestimulation of a target to enhance the analgesic effect of rTMS could be experimented. (4) In addition to stimulating the target site, special stimulation coils can be designed for the deep brain structures involved in pain information processing, such as anterior cingulate cortex (ACC). (5) Clinical trials of non-M1 targets are mostly small samples with insufficient evidence reliability and an older publication year. Therefore, in future clinical studies, it is necessary to conduct large-scale, multicenter, randomized placebo-controlled trials while establishing safe and effective stimulation parameters, selecting homogenous subjects, and reporting the treatment plan with detail and clarity. (6) When multiple SRs in an evidence mapping overlap in the inclusion of primary studies, it may be necessary to cross-check these SRs to determine whether the reported conclusions are the same as well as the extent of the overlap and the impact of the quality of SRs on the applicability of the conclusions. For example, 7 SRs categorized as second PICOs (DLPFC,10 Hz, long sessions) had different findings, with one in each of the categories of “potentially better,” “mixed,” “no difference,” and “potentially worse.” Future reviews could integrate the studies included in all 7 SRs and form new findings. (7) PICOs with multiple bubbles, and particularly those drawing mixed conclusions, may be an area where SRs need to be updated.

The quality of SRs is also an important consideration when conducting SRs. Assessment in this field suggested room for improving SR quality. Future SRs should place more emphasis on the following domains to improve the quality of studies and the validity of the results: reporting explicit statements about the description of the methodology should be designed prior to the conduction of the review; any significant deviations from the protocol should be justified to explain the selection of the study designs for inclusion in the review; a list of excluded studies should be provided and the exclusions should be justified; sources of funding or support for the individual studies included in the SRs should be indicated; and the effect of the risk of bias in individual studies on the total effect should be interpreted and discussed.

### 4.1. Limitations of the Study

Certain limitations in this evidence mapping should be taken into account. First, our SRs search was done in 2021. However, respective study searches were conducted in 2020 or earlier. Thus, studies that were newly published but may not be included in the SRs may have been overlooked. Nevertheless, we believe that these limitations do not substantially impact our results. Secondly, several different types of studies in SRs comparing therapeutic interventions for NP were included. Although most trials were RCTs, observational, open-label, and cohort studies as well as some case reports were also available. Furthermore, the methodologies of some SRs had limitations, and their conclusions can be subject to bias. Therefore, when multiple reviews reach the same conclusion, the conclusion should be explained carefully. Nevertheless, these are reported in detail in our results, and each conclusion can be assessed by the reader, including the limitations of the SRs. Another limitation was found in the selection of studies published solely in the English language, which limited the scope of the evidence mapping.

## 5. Conclusion

NP is a complex and refractory group of diseases. This evidence mapping could encourage clinical workers in the fields of pain, neurology, psychiatry, anesthesiology, and rehabilitation to pay more attention to individual patient characteristics and target the relevant brain regions. The small number of clinical trials in the area of non-M1 target therapy for NP is noteworthy, but the most important clinical issues have been covered as a result of evidence mapping. Evidence mapping is a useful and reliable method to identify currently available research as a suggestion for future research. In the future, more research effort is needed in order to highlight the optimal stimulation protocols and standardize all parameters to fill evidence gaps. More homogenous groups of participants should also be considered. Meanwhile, further efforts are needed to improve the methodological quality and reporting process of SRs.

## Figures and Tables

**Figure 4:**
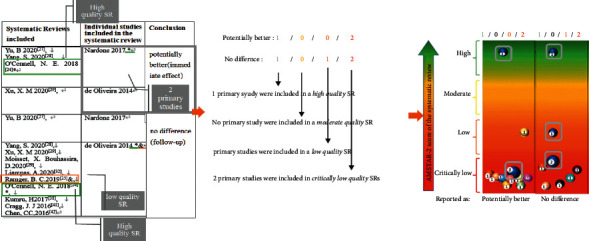
Interpretation of evidence mapping.

**Table 1:** Characteristics of included systematic reviews.

Author and year	Study design	Search date	Objective	Number of studies included	Design and number of included studies	Participants (*n*)
Yu et al., 2020 [28]	SRM	January 2019	To investigate the effect of non-invasive brain stimulation for SCI	11	RCT: 11	274
Yang et al., 2020 [29]	SR	June 2019	To explore the effect of rTMS on different types of pain	106	RCT: 69; OLT: 16; CR: 21	3,264
Xu et al., 2020 [30]	SR	August 2020	To assess the efficacy and safety of non-pharmacological therapies for CPSP	11	RCT: 11	210
Moisset et al., 2020 [31]	SRM	July 2020	To investigate the efficacy of neurostimulation techniques in migraine	38	RCT: 38	2,899
Moisset et al., 2020 [32]	SR	August 2019	To propose all the alternative treatment options for NP	131	RCT: 131	15,776
Liampas et al., 2020 [33]	SRM	November 2019	To describe the prevalence and characteristics of CPSP and investigate the relevant management methods	69	NR	NA
Gatzinsky et al., 2020 [22]	SR	June 2019	To review the efficacy and safety of rTMS on M1	32	RCT: 24; CS: 8	682 (RCT)
Aamir et al., 2020 [34]	SR	June 2019	To evaluate the effect of rTMSfor peripheral NP	12	RCT: 5; CS: 2; CR: 5	188
Stilling et al., 2020 [35]	SR	September 2018	To review the use of TMS and tDCS for specific headache disorders	34	Randomized trials: 20; NRC/prospective cohort/OLT: 14	1,787
Ramger et al., 2019 [26]	SR	2018	To evaluate the efficacy of rTMS and tDCS for CPSP	6	RCT: 1; prospective cohort: 1; CS: 2; cross-over: 2	109
Hamid et al., 2019 [36]	SR	2018	To explore the effect of rTMS on chronic refractory pain, especially in adults with central NP	12	RCT: 12	350
Feng et al., 2019 [37]	SRM	September 2018	To evaluate the efficacy of rTMS and tDCS for migraine	9	RCT: 9	276
O'Connell et al., 2018 [23]	SRM	October 2017	To assess the efficacy of non-invasive cortical stimulation techniques on chronic pain	94	RCT: 94	2,983
Herrero babiloni et al., 2018 [27]	SR	NR	To explore the effect of TMS and tDCS for chronic OFP	14	RCT: 14	228
Lan et al., 2017 [38]	SRM	April 2017	To explore the efficacy of TMS for migraine	5	RCT: 5	313
Kumru et al., 2017 [39]	SR	August 2015	To assess the role of rTMS or peripheral magnetic stimulation for NP	39	NR	892
Goudra et al., 2017 [40]	SRM	NR	To evaluate the effect of rTMS for chronic pain	9	RCT: 6; prospective observational: 3	183
Shirahige et al., 2016	SRM	November 11, 2015, to January 15, 2016	To assess the effect of NIBS on migraine patients	8	RCT: 8	296
Cragg et al., 2016 [42]	SRM	May 2015	To explore the predictors of placebo responses in central NP clinical trials	39	RCT: 39	1,153
Chen et al., 2016 [43]	SR	September 2015	To evaluate the antalgic effects of non-invasive physical modalities on CPSP	16	NA	184
Jin et al., 2015 [44]	SRM	December 2014	To evaluate the optimal parameters of rTMS for NP	25	RCT: 20; self-controlled: 5	589
Galhardoni et al., 2015 [45]	SR	2014	To review the studies on the analgesic effects of rTMS in chronic pain	33	RCT: 33	842
Leung et al., 2009 [46]	SRM	August 2007	To evaluate the overall effect of rTMS for NP and evaluate the effect of treatment parameters.	5	RCT: 5	149

SRs: systematic reviews (with or without meta-analysis); SR: systematic review; SRM: systematic review with meta-analysis; NP: neuropathic pain; SCI: spinal cord injury; rTMS: repetitive transcranial magnetic stimulation; CPSP: central poststroke pain; M1: motor cortex; TMS: transcranial magnetic stimulation; tDCS: transcranial direct current stimulation; OFP: orofacial pain; NIBS: non-invasive brain stimulation; RCT: randomized controlled trial; OLT: open-label trial; CR: case report; NR: not reported; CS: case series; and NA: not available.

**Table 2:** PICOs included in systematic reviews.

PICOs number	PICOs in bubble chart	Stimulation site	Frequency (Hz)	Session schedule	Comparison	Population	Outcomes	Systematic reviews included	Individual studies included in the systematic review				Conclusion
									Controlled trial (parallel)	Controlled trial (cross)	Number of studies	Number of SRs involving the Quality (high/moderate/low/critically low) of Individual studies:	
1	DLPFC, 20 Hz, long sessions vs. sham rTMS	Left DLPFC	20 Hz	12 sessions	Sham	Migraine	Headache index	Yang [29], Stilling [35], Feng [37], Lan [38], Shirahige [41]	Brighina 2004		1	0/0/0/1	Mixed
2	DLPFC, 10 Hz, long sessions vs. sham rTMS	Left DLPFC	10 Hz	23 sessions	Sham	Migraine	MIDAS	Stilling [35]	Conforto 2014		1	0/0/0/1	Potentially worse: immediately
		Left DLPFC	10 Hz	23 sessions	Sham	Migraine	MIDAS	Yang [29], Stilling [35], Hamid [36], Feng [37], Lan [38], Shirahige [41]	Conforto 2014		1	0/0/0/1	No difference: follow-up at 8 weeks
		Left DLPFC	10 Hz	15 sessions	Sham	Chronic widespread pain	NRS	Hamid et al. [36], O'Connell [23]^*∗*^	Avery 2015 ^*∗*^		1	1/0/0/1	Mixed
		Left DLPFC	10 Hz	12 sessions	Standard pharmacotherapy	Migraine	VAS	Yang [29], Stilling [35], Feng [37]	Rapinesi 2016		1	0/0/0/1	Potentially better
3	DLPFC, 10 Hz, medium sessions vs. sham rTMS	Left DLPFC	10 Hz	10 sessions	Sham	BMS	VAS	Yang [29], O'Connell [23] ^*∗*^, Herrero Babiloni [27] &	Umezaki 2016 ^*∗*^ &		1	1/0/1/1	Mixed
4	DLPFC, 10 Hz, short sessions vs. sham rTMS	Left DLPFC	10 Hz	4 sessions	Sham	Mild traumatic brain injury related headache	NRS	Yang [29], Stilling [35]	Leung 2018		1	0/0/0/1	Potentially better: at 1 and 4 weeks
		Left DLPFC	10 Hz	2 rTMS sessions, 1 rTMS + 1 sham	Sham	Postsurgical pain	VAS, morphine use	Yang [29]	Borckardt 2014		1	0/0/0/1	No difference
5	DLPFC, 5 Hz, medium sessions vs. sham rTMS	Left DLPFC	5 Hz	10 sessions		Migraine	MIDAS	Yang [29], Moisset [31]		Sahu 2019	1	0/0/0/1	Potentially better
6	DLPFC, 5 Hz, short sessions vs. sham rTMS	Left DLPFC	5 Hz	5 sessions	Sham	Episodic migraine	Attack frequency	Moisset [31]	Amin 2020		1	0/0/0/1	Potentially better
7	S2,1 Hz, medium sessions vs. sham rTMS	S2	1 Hz	10 sessions	Sham	Chronic visceral pain (visceral pain due to chronic pancreatitis)	VAS	Yang [29], Hamid [36], O'Connell [23]^*∗*^, Galhardoni [45]	Fregni 2011 ^*∗*^		1	1/0/0/1	Mixed
8	S2,1 Hz or 20 Hz, short sessions vs. sham rTMS	S2	1 Hz or 20 Hz	1 session	Sham	Chronic pancreatitis pain	VAS	O'Connell [23]^*∗*^		Fregni 2005^*∗*^	1	1/0/0/0	Unclear
9	Vertex,10 Hz, medium sessions vs. sham rTMS	Vertex	10 Hz	10 sessions	Sham	SCI		Galhardoni et al. [45]	Yılmaz 2014		1	0/0/0/1	No difference
10	Vertex,1 Hz, short sessions vs. sham rTMS	Vertex	1 Hz	5 sessions	Sham	Migraine	NRS	Yang [29], Stilling [35], Moisset [31], Feng [37], Shirahige [41]	Teepker 2010		1	0/0/0/1	No difference
11	PFC, 10 Hz, short sessions vs. sham rTMS	Left PFC	10 Hz	3 sessions	Sham	Intractable neuropathic pain of various origins	NRS	Yang [29], Hamid [36], O'Connell [23]^*∗*^, Herrero Babiloni&, Kumru [39]		Borckardt 2009^*∗*^,&	2	1/0/1/2	Mixed
		Left PFC	10 Hz	1 session	Sham	Postsurgical pain	VAS	Yang [29], Goudra [40], Galhardoni [45]	Borckardt 2006				Mixed
		Left PFC	10 Hz	1 session	Sham	Postsurgical pain	VAS	Yang [29]	Borckardt 2008		1	0/0/0/1	Potentially better
12	S2 vs. S1/M1 vs. sham rTMS, 10 Hz, short sessions	Right S2	10 Hz	3 sessions	S1/M1 and sham	Non-specified orofacial pain, trigeminal neuropathic pain	NRS	Yang [29] Herrero Babiloni [27]&, Kumru [39]		Lindholm 2015&	1	0/0/1/1	Potentially better (S2)
13	PMC/DLPFC vs. sham rTMS, 10 Hz, medium sessions	Left PMC/DLPFC	10 Hz	10 sessions	Sham	SCI	Pain intensity: VAS	Yu [28], Yang [29], O'Connell [23]^*∗*^	Nardone 2017 ^*∗*^		2	1/0/0/2	Potentially better (immediate effect)
		Left PMC/DLPFC	10 Hz	10 sessions	Sham	CPSP	VAS	Xu [30]	de Oliveira 2014				Potentially better (immediate effect)
		Left PMC/DLPFC	10 Hz	10 sessions	Sham	SCI	Pain intensity: VAS	Yu [28]	Nardone 2017		2	1/0/1/2	No difference (follow-up)
		Left PMC/DLPFC	10 Hz	10 sessions	Sham	CPSP	VAS	Yang [29], Xu [30], Moisset and Bouhassira [30], Liampas [33], Ramger [26]&, O'Connell [23]^*∗*^, Kumru [39], Cragg [42], Chen [43]	de Oliveira 2014^*∗*^, &				No difference (from D1 to W4)
14	Left frontal cortex vs. sham rTMS, 10 Hz, short sessions	Left frontal cortex	10 Hz	3 sessions	Sham	Migraine	VAS	Yang [29], Lan [38]	Misra 2013		1	0/0/0/1	Potentially better
15	Over the superior trapezius muscle vs. sham rTMS, 3 Hz/15 Hz, 10 sessions	Over the superior trapezius muscle	3 Hz/15 Hz	10 sessions	Sham	Brachial plexopathy	VAS	Aamir [34], Kumru [39]	Khedr 2012		1	0/0/0/1	Potentially better(1 month)
16	ACC vs. PSI vs. sham rTMS, 10 Hz, 16 sessions	ACC vs. PSI	10 Hz	16 sessions	Sham	CPSP or SCI	NRS	Yang [29], Moisset and Bouhassira [32]	Galhardoni 2019		1	0/0/0/1	No difference
17	S1, SMA, preM vs. M1 vs. sham rTMS, 5 Hz, short sessions	S1, SMA, preM	5 Hz	4 sessions	M1 and sham	NP	VAS	Yang [29], Gatzinsky [22], O'Connell [23]^*∗*^, Kumru [39], Chen [43], Jin [44], Galhardoni [45], Leung [46]		Hirayama 2006^*∗*^	1	1/0/0/1	Mixed
		S1, SMA, preM	5 Hz	2 sessions	M1 and sham	CPSP, SCI, TGNI, PNI, RA	VAS, SF-MPQ	Kumru [39]	Saitoh 2006		1	0/0/0/1	Potentially better (M1, maintained 3 hours)

PICO: population, intervention, control group, outcome; rTMS: repetitive transcranial magnetic stimulation; DLPFC: dorsolateral prefrontal cortex; S2: secondary somatosensory cortex; PFC: prefrontal cortex; PMC: premotor cortex; ACC: anterior cingulate cortex; PSI: posterior superior insula; S1: postcentral gyrus; SMA: supplementary motor area; preM: premotor area. BMS: burning mouth syndrome; SCI: spinal cord injury; CPSP: central poststroke pain; NP: neuropathic pain; TGNI: trigeminal neuropathic pain; PNI: peripheral nerve injury; RA: root avulsion. MIDAS: migraine disability assessment; NRS: numerical rating scale; VAS: visual analog scale; and SF-MPQ: short form of the McGill pain questionnaire. Note: (i) short: 1–5 sessions, medium: 5–10 sessions, and long: >10 sessions. (ii) In the included SRs: high-quality SRs are marked as ^*∗*^; ^&^low-quality SRs; and the rest are critically low-quality SRs; (iii) in the primary studies included in SRs: ^*∗*^included by high- and critically low-quality SRs, and included by low- and critically low-quality SRs; (iv) in the number of SRs involving the quality (high/moderate/low/critically low) of primary studies): taking the 13th PICO (PMC/DLPFC vs. sham rTMS, 10 Hz, medium sessions), as an example, a total of 2 primary studies were involved. The meaning of 1/0/0/2 and 1/0/1/2 is shown below in Figure 4.

## Data Availability

The data generated in this study can be obtained in the supplementary materials provided.

## Supplementary Materials

Supplementary Materials Supplementary Material 1: Database search strategies. Supplementary Material 2: SRs excluded. Supplementary Material 3: AMSTAR-2 assessment. Supplementary Material 4: PICOs' characteristics in the SRs.
