# Supplementary material for: Repetitive Transcranial Magnetic Stimulation for Neuropathic Pain on the Non-Motor Cortex: An Evidence Mapping of Systematic Reviews
Source: Evid Based Complement Alternat Med. 2021 Oct 29;2021:3671800. doi: 10.1155/2021/3671800 (PMC8570850; doi:10.1155/2021/3671800)
Supplement: Supplementary Materials — Supplementary Material 1: Database search strategies. Supplementary Material 2: SRs excluded. Supplementary Material 3: AMSTAR-2 assessment. Supplementary Material 4: PICOs' characteristics in the SRs. [file 3671800.f1.zip › 3671800.f1/SM-1.docx]

**Supplementary material 1: Search strategy**

| **Database** | **Date** | **Terms** | **Results** |
| --- | --- | --- | --- |
| **MEDLINE**  **PubMed** | **2021-1-23** | **#1: Transcranial Magnetic Stimulation** |  |
|  |  | ("Transcranial Magnetic Stimulation"[Mesh]) OR ((((((((((Transcranial Magnetic Stimulation[Title/Abstract]) OR (Magnetic Stimulation, Transcranial[Title/Abstract])) OR (Magnetic Stimulations, Transcranial[Title/Abstract])) OR (Stimulation, Transcranial Magnetic[Title/Abstract])) OR (Stimulations, Transcranial Magnetic[Title/Abstract])) OR (Transcranial Magnetic Stimulations[Title/Abstract])) OR (Transcranial Magnetic Stimulation, Single Pulse[Title/Abstract])) OR (Transcranial Magnetic Stimulation, Paired Pulse[Title/Abstract])) OR (Transcranial Magnetic Stimulation, Repetitive[Title/Abstract])) OR (TMS[Title/Abstract])) | 23473 |
|  |  | **#2： Neuropathic Pain** |  |
|  |  | ("Neuralgia"[Mesh]) OR (((((((((((((((((((((((((((((((((((((((((((((((((Neuralgia[Title/Abstract]) OR (Neuralgias[Title/Abstract])) OR (Neuropathic Pain[Title/Abstract])) OR (Neuropathic Pains[Title/Abstract])) OR (Pain, Neuropathic[Title/Abstract])) OR (Pains, Neuropathic[Title/Abstract])) OR (Neurodynia[Title/Abstract])) OR (Neurodynias[Title/Abstract])) OR (Neuralgia, Atypical[Title/Abstract])) OR (Atypical Neuralgia[Title/Abstract])) OR (Atypical Neuralgias[Title/Abstract])) OR (Neuralgias, Atypical[Title/Abstract])) OR (Neuralgia, Iliohypogastric Nerve[Title/Abstract])) OR (Iliohypogastric Nerve Neuralgia[Title/Abstract])) OR (Iliohypogastric Nerve Neuralgias[Title/Abstract])) OR (Nerve Neuralgia, Iliohypogastric[Title/Abstract])) OR (Nerve Neuralgias, Iliohypogastric[Title/Abstract])) OR (Neuralgias, Iliohypogastric Nerve[Title/Abstract])) OR (Paroxysmal Nerve Pain[Title/Abstract])) OR (Nerve Pain, Paroxysmal[Title/Abstract])) OR (Nerve Pains, Paroxysmal[Title/Abstract])) OR (Pain, Paroxysmal Nerve[Title/Abstract])) OR (Pains, Paroxysmal Nerve[Title/Abstract])) OR (Paroxysmal Nerve Pains[Title/Abstract])) OR (Neuralgia, Perineal[Title/Abstract])) OR (Neuralgias, Perineal[Title/Abstract])) OR (Perineal Neuralgia[Title/Abstract])) OR (Perineal Neuralgias[Title/Abstract])) OR (Neuralgia, Stump[Title/Abstract])) OR (Neuralgias, Stump[Title/Abstract])) OR (Stump Neuralgia[Title/Abstract])) OR (Stump Neuralgias[Title/Abstract])) OR (Neuralgia, Supraorbital[Title/Abstract])) OR (Neuralgias, Supraorbital[Title/Abstract])) OR (Supraorbital Neuralgia[Title/Abstract])) OR (Supraorbital Neuralgias[Title/Abstract])) OR (Neuralgia, Vidian[Title/Abstract])) OR (Neuralgias, Vidian[Title/Abstract])) OR (Vidian Neuralgia[Title/Abstract])) OR (Vidian Neuralgias[Title/Abstract])) OR (Nerve Pain[Title/Abstract])) OR (Nerve Pains[Title/Abstract])) OR (Pain, Nerve[Title/Abstract])) OR (Pains, Nerve[Title/Abstract])) OR (Neuralgia, Ilioinguinal[Title/Abstract])) OR (Ilioinguinal Neuralgia[Title/Abstract])) OR (Ilioinguinal Neuralgias[Title/Abstract])) OR (Neuralgias, Ilioinguinal[Title/Abstract])) OR (NP[Title/Abstract])) | 123428 |
|  |  | #1 AND#2 | 387 |
|  |  | Filters applied: Meta-Analysis, Systematic Review. | 32 |
| **The Cochrane library** | **2021-1-23** | **Neuropathic Pain** |  |
|  |  | #1 MeSH descriptor: [Neuralgia] explode all trees 1685  #2 (Neuralgia):ti,ab,kw OR (Neuralgias):ti,ab,kw OR (Neuropathic Pain):ti,ab,kw OR (Neuropathic Pains):ti,ab,kw OR (Pain, Neuropathic):ti,ab,kw (Word variations have been searched) 5555  #3 (Pains, Neuropathic):ti,ab,kw OR (Neurodynia):ti,ab,kw OR (Neurodynias):ti,ab,kw OR (Neuralgia, Atypical):ti,ab,kw OR (Atypical Neuralgia):ti,ab,kw (Word variations have been searched) 3792  #4 (Atypical Neuralgias):ti,ab,kw OR (Neuralgias, Atypical):ti,ab,kw OR (Neuralgia, Iliohypogastric Nerve):ti,ab,kw OR (Iliohypogastric Nerve Neuralgia):ti,ab,kw OR (Iliohypogastric Nerve Neuralgias):ti,ab,kw (Word variations have been searched) 23  #5 (Nerve Neuralgia, Iliohypogastric):ti,ab,kw OR (Nerve Neuralgias, Iliohypogastric):ti,ab,kw OR (Neuralgias, Iliohypogastric Nerve):ti,ab,kw OR (Paroxysmal Nerve Pain):ti,ab,kw OR (Nerve Pain, Paroxysmal):ti,ab,kw (Word variations have been searched) 39  #6 (Nerve Pains, Paroxysmal):ti,ab,kw OR (Pain, Paroxysmal Nerve):ti,ab,kw OR (Pains, Paroxysmal Nerve):ti,ab,kw OR (Paroxysmal Nerve Pains):ti,ab,kw OR (Neuralgia, Perineal):ti,ab,kw (Word variations have been searched) 43  #7 (Neuralgias, Perineal):ti,ab,kw OR (Perineal Neuralgia):ti,ab,kw OR (Perineal Neuralgias):ti,ab,kw OR (Neuralgia, Stump):ti,ab,kw OR (Neuralgias, Stump):ti,ab,kw (Word variations have been searched) 17  #8 (Stump Neuralgia):ti,ab,kw OR (Stump Neuralgias):ti,ab,kw OR (Neuralgia, Supraorbital):ti,ab,kw OR (Neuralgias, Supraorbital):ti,ab,kw OR (Supraorbital Neuralgia):ti,ab,kw (Word variations have been searched) 18  #9 (Supraorbital Neuralgias):ti,ab,kw OR (Neuralgia, Vidian):ti,ab,kw OR (Neuralgias, Vidian):ti,ab,kw OR (Vidian Neuralgia):ti,ab,kw OR (Vidian Neuralgias):ti,ab,kw (Word variations have been searched) 12  #10 (Nerve Pain):ti,ab,kw OR (Nerve Pains):ti,ab,kw OR (Pain, Nerve):ti,ab,kw OR (Pains, Nerve):ti,ab,kw OR (Neuralgia, Ilioinguinal):ti,ab,kw (Word variations have been searched) 14812  #11 (Ilioinguinal Neuralgia):ti,ab,kw OR (Ilioinguinal Neuralgias):ti,ab,kw OR (Neuralgias, Ilioinguinal):ti,ab,kw OR (NP):ti,ab,kw (Word variations have been searched) 2066  **#12: #1 or #2 or #3 or #4 or #5 or #6 or #7 or #8 or #9 or #10 or #11** | 21380 |
|  |  | **Transcranial Magnetic Stimulation** |  |
|  |  | MeSH descriptor: [Transcranial Magnetic Stimulation] explode all trees 1354  #40 (Transcranial Magnetic Stimulation):ti,ab,kw OR (Magnetic Stimulation, Transcranial):ti,ab,kw OR (Magnetic Stimulations, Transcranial):ti,ab,kw OR (Stimulation, Transcranial Magnetic):ti,ab,kw OR (Stimulations, Transcranial Magnetic):ti,ab,kw (Word variations have been searched) 5265  #41 (Transcranial Magnetic Stimulations):ti,ab,kw OR (Transcranial Magnetic Stimulation, Single Pulse):ti,ab,kw OR (Transcranial Magnetic Stimulation, Paired Pulse):ti,ab,kw OR (Transcranial Magnetic Stimulation, Repetitive):ti,ab,kw OR (TMS):ti,ab,kw (Word variations have been searched)  **#42：#39 or #40 or #41** | 6171 |
|  |  | #12 and #42 | 207 |
|  |  | Filters applied: Meta-Analysis, Systematic Review | 3 |
| **Embase** | **2021-1-23** | **Neuropathic Pain** |  |
|  |  | **#1：**'neuralgia'/exp OR 'neuralgia':ti,ab OR 'neuralgias':ti,ab OR 'neuropathic pain':ti,ab OR 'neuropathic pains':ti,ab OR 'pain, neuropathic':ti,ab OR 'pains, neuropathic':ti,ab OR 'neurodynia':ti,ab OR 'neurodynias':ti,ab OR 'neuralgia, atypical':ti,ab OR 'atypical neuralgia':ti,ab OR 'atypical neuralgias':ti,ab OR 'neuralgias, atypical':ti,ab OR 'neuralgia, iliohypogastric nerve':ti,ab OR 'iliohypogastric nerve neuralgia':ti,ab OR 'iliohypogastric nerve neuralgias':ti,ab OR 'nerve neuralgia, iliohypogastric':ti,ab OR 'nerve neuralgias, iliohypogastric':ti,ab OR 'neuralgias, iliohypogastric nerve':ti,ab OR 'paroxysmal nerve pain':ti,ab OR 'nerve pain, paroxysmal':ti,ab OR 'nerve pains, paroxysmal':ti,ab OR 'pain, paroxysmal nerve':ti,ab OR 'pains, paroxysmal nerve':ti,ab OR 'paroxysmal nerve pains':ti,ab OR 'neuralgia, perineal':ti,ab OR 'neuralgias, perineal':ti,ab OR 'perineal neuralgia':ti,ab OR 'perineal neuralgias':ti,ab OR 'neuralgia, stump':ti,ab OR 'neuralgias, stump':ti,ab OR 'pains, nerve':ti,ab OR 'pain, nerve':ti,ab OR 'nerve pains':ti,ab OR 'nerve pain':ti,ab OR 'vidian neuralgias':ti,ab OR 'vidian neuralgia':ti,ab OR 'neuralgias, vidian':ti,ab OR 'neuralgia, vidian':ti,ab OR 'supraorbital neuralgias':ti,ab OR 'supraorbital neuralgia':ti,ab OR 'neuralgias, supraorbital':ti,ab OR 'neuralgia, supraorbital':ti,ab OR 'stump neuralgias':ti,ab OR 'stump neuralgia':ti,ab OR 'neuralgia, ilioinguinal':ti,ab OR 'ilioinguinal neuralgia':ti,ab OR 'ilioinguinal neuralgias':ti,ab OR 'neuralgias, ilioinguinal':ti,ab OR 'np':ti,ab | 172589 |
|  |  | **transcranial magnetic stimulation** |  |
|  |  | #13. 'tms':ti,ab  #12. 'transcranial magnetic stimulation, repetitive':ti,ab  #11. 'transcranial magnetic stimulation, paired pulse':ti,ab  #10. 'transcranial magnetic stimulation, single pulse':ti,ab  #9. 'transcranial magnetic stimulations':ti,ab  #8. 'stimulations, transcranial magnetic':ti,ab  #7. 'stimulation, transcranial magnetic':ti,ab  #6. 'magnetic stimulations, transcranial':ti,ab  #5. 'magnetic stimulation, transcranial':ti,ab  #4. 'transcranial magnetic stimulation':ti,ab  #3. 'transcranial magnetic stimulation'/exp  **#14：#3 OR #4 OR #5 OR #6 OR #7 OR #8 OR #9 OR #10 OR #11 OR #12 OR #13** | 33879 |
|  |  | #1 AND #14 | 602 |
|  |  | Filters applied: Meta-Analysis, Systematic Review | 49 |
| **Epistemonikos** | **2021-1-23** | **Transcranial Magnetic Stimulation** |  |
|  |  | **#1:** (title:(Transcranial Magnetic Stimulation) OR abstract:(Transcranial Magnetic Stimulation)) OR (title:(Magnetic Stimulation, Transcranial) OR abstract:(Magnetic Stimulation, Transcranial)) OR (title:(Magnetic Stimulations, Transcranial) OR abstract:(Magnetic Stimulations, Transcranial)) OR (title:(Stimulation, Transcranial Magnetic) OR abstract:(Stimulation, Transcranial Magnetic)) OR (title:(Stimulations, Transcranial Magnetic) OR abstract:(Stimulations, Transcranial Magnetic)) OR (title:(Transcranial Magnetic Stimulations) OR abstract:(Transcranial Magnetic Stimulations)) OR (title:(Transcranial Magnetic Stimulation, Single Pulse) OR abstract:(Transcranial Magnetic Stimulation, Single Pulse)) OR (title:(Transcranial Magnetic Stimulation, Paired Pulse) OR abstract:(Transcranial Magnetic Stimulation, Paired Pulse)) OR (title:(Transcranial Magnetic Stimulation, Repetitive) OR abstract:(Transcranial Magnetic Stimulation, Repetitive)) OR (title:(TMS) OR abstract:(TMS)) |  |
|  |  | **Neuropathic Pain** |  |
|  |  | **#2:** (title:(Neuralgia) OR abstract:(Neuralgia)) OR (title:(Neuropathic Pain) OR abstract:(Neuropathic Pain)) OR (title:(Neurodynia) OR abstract:(Neurodynia)) OR (title:(Nerve Pain) OR abstract:(Nerve Pain)) OR (title:(NP) OR abstract:(NP)) |  |
|  |  | **#1 AND #2** Filters applied: Meta-Analysis, Systematic Review | 41 |
