# Supplementary material for: Repetitive Transcranial Magnetic Stimulation for Neuropathic Pain on the Non-Motor Cortex: An Evidence Mapping of Systematic Reviews
Source: Evid Based Complement Alternat Med. 2021 Oct 29;2021:3671800. doi: 10.1155/2021/3671800 (PMC8570850; doi:10.1155/2021/3671800)
Supplement: Supplementary Materials — Supplementary Material 1: Database search strategies. Supplementary Material 2: SRs excluded. Supplementary Material 3: AMSTAR-2 assessment. Supplementary Material 4: PICOs' characteristics in the SRs. [file 3671800.f1.zip › 3671800.f1/SM-2.docx]

**Supplementary material 2:**

**studies that were evaluated in full-text and were excluded.**

| N | **Author** | **Year** | **Title** | **Reason for exclusion** |
| --- | --- | --- | --- | --- |
| 1 | Cassani, R. et al | 2020 | Virtual reality and non-invasive brain stimulation for rehabilitation applications: a systematic review | Wrong population:  TMS is not associated with neuropathic pain in the population treated |
| 2 | Young, J. R. et al | 2020 | Non-invasive brain stimulation modalities for the treatment and prevention of opioid use disorder: a systematic review of the literature | Wrong population:  study on opioid use disorder; |
| 3 | Treister, A. K et al | 2017 | Demystifying Poststroke Pain: From Etiology to Treatment | No appropriate control group |
| 4 | Zucchella, C.et al | 2020 | Non-invasive Brain and Spinal Stimulation for Pain and Related Symptoms in Multiple Sclerosis: A Systematic Review | Wrong stimulation site |
| 5 | Zeng, H.et al | 2020 | Non-invasive neuromodulation effects on painful diabetic peripheral neuropathy: a systematic review and meta-analysis | Wrong stimulation site |
| 6 | Shen, Z. et al | 2020 | Effect of non-invasive brain stimulation on neuropathic pain following spinal cord injury: A systematic review and meta-analysis | Wrong stimulation site |
| 7 | Pacheco-Barrios, K. | 2020 | Neuromodulation Techniques in Phantom Limb Pain: A Systematic Review and Meta-analysis | Wrong stimulation site |
| 8 | Liampas, A.  Rekatsina, M et al | 2020 | Non-Pharmacological Management of Painful Peripheral Neuropathies: A Systematic Review | Wrong stimulation site |
| 9 | Chang, M. C.et al | 2020 | The effect of rTMS in the management of pain associated with CRPS | Wrong stimulation site |
| 10 | Cardenas-Rojas, A.et al | 2020 | Noninvasive brain stimulation combined with exercise in chronic pain: a systematic review and meta-analysis | Wrong stimulation site |
| 11 | Nardone, R. et al | 2019 | Transcranial magnetic stimulation in subjects with phantom pain and non-painful phantom sensations: A systematic review | Wrong stimulation site |
| 12 | Akyuz, G. et al | 2019 | Noninvasive neuromodulation techniques for the management of phantom limb pain: a systematic review of randomized controlled trials | Wrong stimulation site |
| 13 | Gao, F.et al | 2017 | Repetitive transcranial magnetic stimulation for pain after spinal cord injury: a systematic review and meta-analysis | Wrong stimulation site |
| 14 | Mulla, S. M. et al | 2015 | Management of Central Poststroke Pain: Systematic Review of Randomized Controlled Trials | Wrong stimulation site |
| 15 | Moreno-Duarte, I. et al | 2014 | Targeted therapies using electrical and magnetic neural stimulation for the treatment of chronic pain in spinal cord injury | Wrong stimulation site |
| 16 | Boldt, I. et al | 2014 | Non‐pharmacological interventions for chronic pain in people with spinal cord injury | Wrong stimulation site |
| 17 | Mehta, S. et al | 2013 | Neuropathic pain post spinal cord injury part 1: systematic review of physical and behavioral treatment | Wrong stimulation site |
| 18 | Cossins, L. et al | 2013 | Treatment of complex regional pain syndrome in adults: a systematic review of randomized controlled trials published from June 2000 to February 2012 | Wrong stimulation site |
| 19 | Zaghi, S. et al | 2011 | Assessment and treatment of pain with non-invasive cortical stimulation | Wrong stimulation site |
| 20 | Kumar, B. et al | 2009 | Central poststroke pain: A review of pathophysiology and treatment | Wrong stimulation site |
| 21 | Reuter, U. et al | 2019 | Non-invasive neuromodulation for migraine and cluster headache: a systematic review of clinical trials | Not rTMS |
| 22 | Di Pietro, F.et al | 2013 | Primary motor cortex function in complex regional pain syndrome: A systematic review and meta-analysis | No pain outcome:  Study Assesses Changes in Primary Motor Cortex in Complex Regional Pain Syndrome by Several Neuroimaging Techniques |
| 23 | Nardone, R. et al | 2018 | Transcranial magnetic stimulation studies in complex regional pain syndrome type I: A review | No pain outcome  Study NP-related pathophysiology |
| 24 | Reuter, U. et al | 2019 | Non-invasive neuromodulation for migraine and cluster headache: a systematic review of clinical trials | No pain outcome:  aim to assess the scientific rigour and clinical relevance of these  devices and their associated clinical data. |
| 25 | Nardone, R. et al | 2015 | Descending motor pathways and cortical physiology after spinal cord injury assessed by transcranial magnetic stimulation: a systematic review | No pain outcome  This study aim to identify neurophysiological biomarkers through TMS technology to help assess the extent of nerve damage, elucidate mechanisms of nerve repair, predict clinical outcomes, and identify therapeutic targets. |
| 26 | Parker, R. S. et al | 2016 | Is Motor Cortical Excitability Altered in People with Chronic Pain? A Systematic Review and Meta-Analysis | No pain outcome  This review studies examining corticospinal and intracortical  excitability using transcranial magnetic stimulation in people with chronic pain compared to healthy controls |
| 27 | Araújo, H. A. et al | 2011 | Systematic literature review on the effects of noninvasive cortical stimulation for chronic pain control | Wrong study design ；Conference abstract |
| 28 | Cruccu, G. et al | 2007 | EFNS guidelines on neurostimulation therapy for neuropathic pain | Wrong study design ；guideline |
| 29 | Cruccu, G. et al | 2016 | EAN guidelines on central neurostimulation therapy in chronic pain conditions | Wrong study design ；guideline |
| 30 | Dosenovic, S. et al | 2017 | Interventions for Neuropathic Pain: An Overview of Systematic Reviews | Wrong study design；  An overview of the study of systematic reviews |
| 31 | Soulia, Vassiliki et al | 2011 | Non-invasive and Non-pharmacological Methods for the Alleviation of Neuropathic Pain | Non-English |
