# Supplementary material for: Repetitive Transcranial Magnetic Stimulation for Neuropathic Pain on the Non-Motor Cortex: An Evidence Mapping of Systematic Reviews
Source: Evid Based Complement Alternat Med. 2021 Oct 29;2021:3671800. doi: 10.1155/2021/3671800 (PMC8570850; doi:10.1155/2021/3671800)
Supplement: Supplementary Materials — Supplementary Material 1: Database search strategies. Supplementary Material 2: SRs excluded. Supplementary Material 3: AMSTAR-2 assessment. Supplementary Material 4: PICOs' characteristics in the SRs. [file 3671800.f1.zip › 3671800.f1/SM-3.pdf]

Items	Content for critically appraising systematic reviews of items	Year of publication	2020									2019			2018		2017				2016		2015		2009
		Serial number	1	2	3	4	5	6	7	8	9	10	11	12	13	14	15	16	17	18	19	20	21	22	23
	Note: "*" are AMSTAR 2 critical domains.	Systematic Reviews included	Yu, B.	Yang, S.	Xu, X. M.	Moisset, X./Pereira, B.	Moisset, X./Bouhassira, D.	Liampas, A./Velidakis, N.	Gatzinsky, K	Aamir, A	Stilling, J. M.	Ramger, B. C.	Hamid, P.	Feng, Y.	O'Connell, N. E.	Herrero Babiloni, A.	Lan, L.	Kumru, H.	Goudra, B.	Shirahige, L.	Cragg, J. J.	Chen, C. C.	Jin, Y.	Galhardoni, R.	Leung, A.
Items	Content for critically appraising systematic reviews of items	Rating overall confidence in the results of the review	Critically low	Critically low	Critically low	Critically low	Critically low	Critically low	Critically low	Critically low	Critically low	Low	Critically low	Critically low	High	Critically low	Critically low	Critically low	Critically low	Critically low	Critically low	Critically low	Critically low	Critically low	Critically low
1	Did the research questions and inclusion criteria for the review include the components of PICO?																								
	I.The authors described population, intervention, comparator group,outcome in detail, and recommend timeframe for follow-up were described selectively.	Yes	√	√	√	√	√	√	√	√	√	√	√	√	√	√	√	√	√	√	√	√	√	√	√
	II.In the review, the author did not fully describe the components of PICO.	No																							
2*	Did the report of the review contain an explicit statement that the review methods were established prior to the conduct of the review and did the report justify any significant deviations from the protocol?																								
	I.The authors stated that they had a written protocol or guide that included ALL the following:review question(s),a search strategy,inclusion/exclusion criteria and a risk of bias assessment.	Partial Yes				√	√																		
	II.As for partial yes, plus the protocol should be registered and should also have specified:a meta-analysis/synthesis plan, if appropriate, and a plan for investigating causes of heterogeneity justification for any deviations from the protocol.	Yes	√					√			√	√			√	√				√					
	III.The authors did not mention the existence of the protocol in the review, and the protocol was not retrieved, and the research method cannot be judged as predetermined based on the description in the text.	No		√	√				√	√			√	√			√	√	√		√	√	√	√	√
3	Did the review authors explain their selection of the study designs for inclusion in the review?																								
	I.The authors explained in detail the rationale for including only RCTs,only NRSI, or both RCTs and NRSI.	Yes					√																		
	II.The authors did not explain the rationale for any types of studies included.	No	√	√	√	√		√	√	√	√	√	√	√	√	√	√	√	√	√	√	√	√	√	√
4*	Did the review authors use a comprehensive literature search strategy?																								
	I.The authors searched at least 2 databases (relevant to research question),provided search terms and/or a search strategy,and justified publication restrictions (eg, language and time).	Partial Yes				√						√	√	√									√	√	√
	II.As for partial yes, plus the review searched the reference lists/bibliographies of included studies,searched trial/study registries, included / consulted content experts in the field,where relevant, searched for grey literature,and conducted search within 24 months of completion of the review.	Yes	√		√		√		√		√				√		√			√		√			
	III.The author only searched a single database,and/or did not provide search terms and search strategies,and/or did not give a reasonable explanation of language, time and other restrictions.	No		√				√		√						√		√	√		√				
5	Did the review authors perform study selection in duplicate?																								
	I.At least two reviewers independently agreed on selection of eligible studies and achieved consensus on which studies to include,or two reviewers selected a sample of eligible studies and achieved good agreement (at least 80 per cent), with the remainder selected by one reviewer.	Yes	√		√			√	√		√	√		√	√	√			√	√			√		√
	II.One reviewer had done selection of eligible studies independently,or the process of selecting studies was not described in the text.	No		√		√	√			√			√				√	√			√	√		√	
6	Did the review authors perform data extraction in duplicate?																								
	I.At least two reviewers achieved consensus on which data to extract or from included studies,or two reviewers extracted data from a sample of eligible studies and achieved good agreement (at least 80 per cent), with the remainder extracted by one reviewer.	Yes	√		√			√	√					√	√	√	√			√					
	II.One reviewer extracted data independently,or the process of extracting data was not described in the text.	No		√		√	√			√	√	√	√					√	√		√	√	√	√	√
7*	Did the review authors provide a list of excluded studies and justify the exclusions?																								
	I.The authors provided a list of all potentially relevant studies that were read in full text form but excluded from the review.	Partial Yes																							
	II.The authors provided a list of all potentially relevant studies that were read in full text form but excluded from the review,and justified the exclusion from the review of each potentially relevant study.	Yes													√										√
	III.The authors did not provide a list of all potentially relevant studies and reasons excluded from the review,which were read in full text form.	No	√	√	√	√	√	√	√	√	√	√	√	√		√	√	√	√	√	√	√	√	√	
8	Did the review authors describe the included studies in adequate detail?																								
	I.The authors described the basic characteristics of the populations, interventions,comparators, outcomes and research designs.	Partial Yes															√		√		√	√	√	√	√
	II.The authors described in detail the basic characteristics of the populations, intervention and comparator (including doses where relevant),study's setting, outcomes, research designs, timeframe for follow-up and so on.	Yes	√	√	√	√	√		√	√	√	√	√	√	√	√		√		√					
	III.The authors did not fully describe the basic characteristics of the included studies.	No						√																	
9*	Did the review authors use a satisfactory technique for assessing the risk of bias (RoB) in individual studies that were included in the review?																								
	RCTs																								
	I.The authors must have selected an appropriate risk of bias(RoB) assessment tool to assess RoB from unconcealed allocation, and lack of blinding of patients and assessors when assessing outcomes (unnecessary foRoBjective outcomes such as all cause mortality)	Partial Yes										√		√									√		
	II.As for partial yes, plus the authors must have selected an appropriate risk of bias(RoB) assessment tool to assess the presence of bias,and must also have assessed RoB from allocation sequence that was not truly random and selection of the reported result from among multiple measurements or analyses of a specified outcome.	Yes			√	√	√	√	√		√		√		√	√	√			√					

	III.Includes only NRSI in the review.	Yes																						
	IV.The authors did not assess the presence of RoB in the included RCT intervention studies, or inappropriately assessed the presence of RoB.	No	√	√						√								√	√		√	√		√	√
	NRSI																							
	I.The authors must have selected an appropriate risk of bias assessment tool to assess RoB from confounding and selection bias.	Partial Yes										√													
	II.As for partial yes, plus the authors must have selected an appropriate risk of bias(RoB) assessment tool to assess the presence of bias,and must also have assessed RoB from methods used to ascertain exposures， outcomes and selection of the reported result from among multiple measurements or analyses of a specified outcome.	Yes						√			√														
	III.Includes only RCTs in the review.	Yes	√		√	√	√		√				√	√	√	√	√			√	√			√	
	IV.The authors did not assess the presence of RoB in the included NRSI intervention studies, or inappropriately assessed the presence of RoB.	No		√						√								√	√			√	√		√
10	Did the review authors report on the sources of funding for the studies included in the review?																								
	I.Must have reported on the sources of funding for individual studies included in the review,or reporting that the reviewers looked for this information but it was not reported by study authors also qualifies.	Yes													√										
	II.The authors did not find, follow, or report information on the funding sources in included study.	No	√	√	√	√	√	√	√	√	√	√	√	√		√	√	√	√	√	√	√	√	√	√
11*	If meta-analysis was performed did the review authors use appropriate methods for statistical combination of results?																								
	I.For RCTs,the authors justified combining the data in a meta-analysis and they used an appropriate weighted technique(eg,effect size and statistical approach) to combine study results and adjusted for heterogeneity if present,and investigated the causes of any heterogeneity.	Yes	√											√			√		√	√					
	II.For NRSI,the authors justified combining the data in a meta-analysis and used an appropriate weighted technique to combine study results, adjusting for heterogeneity if present,and they statistically combined effect estimates from NRSI that were adjusted for confounding, rather than combining raw data, or justified combining raw data when adjusted effect estimates were not available.They reported separate summary estimates for RCTs and NRSI separately when both were included in the review.	Yes													√										
	III.Systematic review as a qualitative study.	No meta-analysis conducted		√	√		√		√	√	√	√	√			√		√				√		√	
	IV.The authors chose an inappropriate statistical approach.	No				√		√													√		√		√
12	If meta-analysis was performed, did the review authors assess the potential impact of RoB in individual studies on the results of the meta-analysis or other evidence synthesis?																								
	I.The authors included only high quality and low risk of bias RCTs.	Yes												√											
	II.If the pooled estimate was based on RCTs and/or NRSI at variable RoB, the authors performed analyses to investigate possible impact of RoB on summary estimates of effect.	Yes													√					√					
	III.Systematic review as a qualitative study.	No meta-analysis conducted		√	√		√		√	√	√	√	√			√		√				√		√	
	IV.The authors did not investigate the effect of the presence of RoB on the total effect.	No	√			√		√									√		√		√		√		√
13*	Did the review authors account for RoB in individual studies when interpreting/discussing the results of the review?																								
	I.The authors included only high quality and low risk of bias RCTs.	Yes																							
	II.If RCTs with moderate or high RoB, or NRSI were included the review provided a discussion of the likely impact of RoB on the results.	Yes			√				√			√			√	√				√					
	III.The authors did not investigate the effect of the presence of RoB on the total effect.	No	√	√		√	√	√		√	√		√	√			√	√	√		√	√	√	√	√
14	Did the review authors provide a satisfactory explanation for, and discussion of, any heterogeneity observed in the results of the review?																								
	I.There was no significant heterogeneity in the results.	Yes	√																√						
	II.If heterogeneity was present the authors performed an investigation of sources of any heterogeneity in the results and discussed the impact of this on the results of the review.	Yes				√		√	√			√	√	√	√	√				√	√			√	√
	III.The authors did not investigate the sources of heterogeneity in the results and/or did not discuss its impact of this on the results of the review.	No		√	√		√			√	√						√	√				√	√		
15*	If they performed quantitative synthesis did the review authors carry out an adequate investigation of publication bias (small study bias) and discuss its likely impact on the results of the review?																								
	I.The authors performed graphical or statistical tests for publication bias and discussed the likelihood and magnitude of impact of publication bias.	Yes	√					√							√				√		√		√		
	II.The authors did not test for publication bias and/or discuss its impact on the results.	No				√								√			√			√					√
	III.Systematic review as a qualitative study.	No meta-analysis conducted		√	√		√		√	√	√	√	√			√		√				√		√	
16	Did the review authors report any potential sources of conflict of interest, including any funding they received for conducting the review?																								
	The authors described their funding sources and reported no conflicts of interest.	Yes	√	√	√	√		√	√		√	√	√		√		√	√	√		√	√	√	√	
	The authors described their funding sources and how they managed potential conflicts of interest.	Yes																							
	The author did not describe the source of funding, and/or declare a conflict of interest relationship.	No					√			√				√		√				√					√
