# Supplementary material for: Repetitive Transcranial Magnetic Stimulation for Neuropathic Pain on the Non-Motor Cortex: An Evidence Mapping of Systematic Reviews
Source: Evid Based Complement Alternat Med. 2021 Oct 29;2021:3671800. doi: 10.1155/2021/3671800 (PMC8570850; doi:10.1155/2021/3671800)
Supplement: Supplementary Materials — Supplementary Material 1: Database search strategies. Supplementary Material 2: SRs excluded. Supplementary Material 3: AMSTAR-2 assessment. Supplementary Material 4: PICOs' characteristics in the SRs. [file 3671800.f1.zip › 3671800.f1/SM-4.pdf]

Number	Systematic Reviews included	Population	Intervention	Comparison	Outcomes	Individual studies included in the systematic review<br>Controlled trial(parallel)	Controlled trial(cross)	OLT	not reported in systen	Conclusion(follow up)
**1**	Yu, B et al<br>2020	SCI	rTMS<br>ositioning: left prefrontal cortex/dorsolateral prefrontal cortex<br>Parameters: using a figure-of-8 coil, 25 trains at 10 Hz for 5 secs, 120% of the resting motor threshold intensity, 1250 pulses per session<br>Sessions: 10 sessions<br>Control: using a sham coil	sham	Pain intensity: VAS	Nardone et al,2017<br>*Double-blinded RCT*				beneficial (immediately after stimulation）
**1**	Yu, B et al<br>2020	SCI	rTMS Positioning: left prefrontal cortex/dorsolateral prefrontal cortex<br>Parameters: using a figure-of-8 coil, 25 trains at 10 Hz for 5 secs, 120% of the resting motor threshold intensity, 1250 pulses per session<br>Sessions: 10 sessions Control: using a sham coil	sham	Pain intensity: VAS	Nardone et al,2017<br>*Double-blinded RCT*				no difference （follow-up)
**2**	Yang, S. et al<br>2020	Intractable neuropathic pain of various origins	5 HZ 90%RMT 50 pulses, 10 trains/session, total 500 pulses/session, ITI = 50 s<br>Figure 8 M1, S1, PMC, SMA<br>4 rTMS sessions		VAS		Hirayama et al,2006			
**2**	Yang, S. et al<br>2020	Intractable neuropathic pain of various origins	rTMS<br>frequency:10HZ<br>intensity:100% RMT<br>parameters and dosage: Total 4,000 pulses/ session, ITI = 20 s<br>coil type:Figure 8<br>stimulation site: Left PFC<br>session schedule: 3 rTMS sessions	sham	NRS		Borckardt et al.,2009 Cross-over			unclear(Not reported）
**2**	Yang, S. et al<br>2020	Intractable neuropathic pain of various origins	rTMS<br>frequency:1HZ<br>intensity:110% RMT<br>parameters and dosage: Total 1,600 pulses/session<br>coil type:NA<br>stimulation site: Right DLPFC<br>session schedule: 15 rTMS sessions	sham	VAS			Sampson et al,2011		potentially better
**2**	Yang, S. et al<br>2020	CPSP	rTMS<br>frequency:10HZ<br>intensity:120% RMT<br>parameters and dosage: 50 pulses, 25 trains/session, total 1,250 pulses, ITI = 25s<br>coil type:Figure 8<br>stimulation site: Left PMC/DLPMC<br>session schedule: 10 rTMS sessions	sham	VAS	de Oliveira et al,2014 *Parallel*				no difference

**2**	Yang, S. et al 2020	SCI	rTMS<br>frequency:10HZ<br>intensity:120% RMT<br>parameters and dosage: 50 pulses, 25 trains, total 1,250 pulses, ITI = 25 s<br>coil type:Figure 8<br>stimulation site: Left PMC/DLPMC<br>session schedule: 10 rTMS sessions		VAS	Nardone et al.,2017<br>Parallel			potentially better(after 2 weeks)
**2**	Yang, S. et al 2020	CPSP OR SCI	ACC-rTMS and PSI-rTMS frequency:10HZ<br>intensity:120% RMT<br>parameters and dosage: 100 pulses × 15 trains/sessions, total 1,500 pulses/session, ITI = 50 s<br>coil type:Figure 8<br>stimulation site: ACC vs. PSI<br>session schedule: 16 rTMS sessions	sham	NRS	Galhardoni et al,2019<br>Parallel			no difference
**2**	Yang, S. et al 2020	Migraine	rTMS frequency:10HZ<br>intensity:90% MT<br>parameters and dosage: 10 trains/session, 2s duration, ITI = 30 s<br>coil type:Figure 8<br>stimulation site: Left DLPFC<br>session schedule: 12 rTMS sessions			Brighina et al,2004<br>Parallel			potentially better
**2**	Yang, S. et al 2020	Migraine	High rTMS frequency:NA<br>intensity:NA<br>parameters and dosage: NA<br>coil type:NA<br>stimulation site: NA<br>session schedule: 3 rTMS sessions	low rTMS	Likert-type scale			Clarke et al,2006	potentially better
**2**	Yang, S. et al 2020	Migraine	TMS frequency:sTMS<br>intensity:NA<br>parameters and dosage: Two pulses 30 s apart, rise time 180μs, total pulse length < 1ms<br>coil type:NA<br>stimulation site: Occiput<br>session schedule: sTMS		Global assessment of pain	Lipton et al,2010<br>Parallel			potentially better
**2**	Yang, S. et al 2020	Migraine	rTMS frequency:1HZ<br>intensity:Visual MT-2%<br>parameters and dosage: 500 pulses, 2 trains/session, total 1,000 pulses, ITI = 60 s<br>coil type:Circular coil<br>stimulation site: Vertex<br>session schedule:5 rTMS sessions		NRS	Teepker et al,2010	Parallel		no difference
**2**	Yang, S. et al 2020	Migraine	rTMS frequency:10HZ<br>intensity:70%MT<br>parameters and dosage: 60 pulses, 10 trains/session, total 600 pulses, ITI = 45 s<br>coil type:Figure 8<br>stimulation site: Left frontal cortex<br>session schedule:3 rTMS sessions	one true and two sham rTMS	VAS	Misra UK, et al,2013<br>Parallel			potentially better

**2**	Yang, S. et al 2020	Migraine	rTMS frequency:10HZ<br>intensity:110% RMT<br>parameters and dosage: 50 pulses, 32 trains/session, 5s duration, total 1,600 pulses/session, ITI = 30 s<br>coil type:Figure 8<br>stimulation site: Left DLPFC<br>session schedule: 23 rTMS sessions	sham	MIDAS	Conforto et al. 2014 Parallel			no difference
**2**	Yang, S. et al 2020	Migraine	TMS frequency:sTMS<br>intensity:NA<br>parameters and dosage: Rise time of 180μs, total pulse length < 1ms<br>coil type:NA<br>stimulation site: Occiput<br>session schedule: sTMS		NRS			Bhola et al,2015	beneficial
**2**	Yang, S. et al 2020	Migraine	rTMS frequency:10HZ<br>intensity:110% RMT<br>parameters and dosage: 50 pulses, 32 trains/session, 5s duration, total 1,600 pulses/session, ITI = 30 s<br>coil type:Figure 8<br>stimulation site: Left DLPFC<br>session schedule: 23 rTMS sessions	standard pharmacotherapy	VAS	Rapinesi et al.2016 Parallel			potentially better
**2**	Yang, S. et al 2020	mild traumatic brain injury related headache (MBTI-HA)	rTMS frequency:10HZ<br>intensity:80% RMT<br>parameters and dosage:100 pulses, 20 trains/session, total 2,000 pulses/session, ITI = 1 s<br>coil type:Figure 8<br>stimulation site: Left DLPFC<br>session schedule: 4 rTMS sessions	sham		Leung et al. 2018 Parallel			potentially better（4-week）
**2**	Yang, S. et al 2020	Migraine	rTMS frequency:5HZ<br>intensity:80% RMT<br>parameters and dosage:30 pulses, 20 trains/session, total 600 pulses/session, ITI = 8 s<br>coil type:Figure 8<br>stimulation site: Left DLPFC<br>session schedule: 10 rTMS sessions		MIDAS		Sahu et al.2019 Cross-over		beneficial
**2**	Yang, S. et al 2020	non-specified orofacial pain	rTMS frequency:10HZ<br>intensity:90% RMT<br>parameters and dosage:Total 1000 pulses/session, ITI = 10 s<br>coil type:Figure 8<br>stimulation site: Contralateral S1/M1 vs right S2<br>session schedule: 3 rTMS sessions at S1/M1, S2	sensorimotor (S1/M1), right secondary somatosensory (S2), and sham stimulations in random order			Lindholm et al. 2015 Cross-over		potentially better（S2)

**2**	Yang, S. et al 2020	burning mouth syndrome (BMS)	rTMS frequency:10HZ<br>intensity:80% RMT<br>parameters and dosage:Total 3,000 pulses/session, ITI = 10 s<br>coil type:Figure 8<br>stimulation site: Left DLPFC<br>session schedule: 10 rTMS sessions	sham		Umezaki et al. 2016<br>*Paralel*	potentially better （after 1 week)
**2**	Yang, S. et al 2020	postsurgical pain	rTMS frequency:10HZ<br>intensity:80% RMT<br>parameters and dosage:Total 3,000 pulses/session, ITI = 10 s<br>coil type:Figure 8<br>stimulation site: Left DLPFC<br>session schedule: Single rTMS session	sham	VAS, morphine use	Borckardt et al. 2006<br>*Parallel*	potentially better
**2**	Yang, S. et al 2020	postsurgical pain	rTMS frequency:10HZ<br>intensity:80% RMT<br>parameters and dosage:Total 4,000 pulses/session, ITI = 20 s<br>coil type:Figure 8<br>stimulation site: Left DLPFC<br>session schedule: Single rTMS session		VAS, morphine use	Borckardt et al. 2008<br>*Parallel*	potentially better
**2**	Yang, S. et al 2020	Chronic visceral pain ：visceral pain due to chronic pancreatitis	rTMS frequency:1HZ<br>intensity:NA<br>parameters and dosage:Total 1,600 pulses/session<br>coil type:Figure 8<br>stimulation site: S2<br>session schedule: 10 rTMS sessions	sham	VAS	Fregni et al. 2011<br>Parallel	potentially better (at least 3 weeks)
**2**	Yang, S. et al 2020	postsurgical pain	rTMS frequency:10HZ<br>intensity:100%RMT<br>parameters and dosage:Total 4,000 pulses/session, ITI = 20 s<br>coil type:Figure 8<br>stimulation site: Left DLPFC<br>session schedule: 2 rTMS sessions, 1 rTMS + 1 sham	two sessions real rTMS (28 patients),<br>two sessions sham (28 patients),<br>real + sham rTMS (27 patients),<br>sham + real rTMS (25 patients)		Borckardt et al.2014<br>*Parallel*	no difference
**3**	Xu, X. M et al 2020	Refractory CPSP	rTMS<br>(left PMC/DLPFC)<br>frequencies：10 Hz;<br>intensity,120% of the RMT;<br>pulses, 25 series of 5-s;<br>10 daily sessions every day<br>Duration：2wk	sham-rTMS	VAS	Oliveira et al,2014<br>*Randomized,double-blinded, sham-controlled trial*	potentially better（immediate effect）

**4**	Moisset, X.et al 2020	migraine	single-pulse<br>transcranial magnetic stimulation (TMS)<br>occiput,<br>sTMS	sham	Migraine Disability Assessment Scale (MIDAS)	*Lipton RB, 2010 randomised, double-blind, parallel-group, sham-controlled trial*		potentially better(after 2 h)
**4**	Moisset, X.et al 2020	migraine	5hz rTMS over the left dorsolateral prefrontal cortex LDLPFC<br>5 rTMS sessions over 1 week, 900 pulses/session, 5Hz, 100% RMT	sham<br>50% of the machine output with perpendicular coil for the Sham group	Attack frequency	Amin R, et al 2020<br>*randomized double-blinded placebo-controlled*		potentially better
**4**	Moisset, X.et al 2020	migraine	active rTMS<br>ten sessions of iTBS over the left DLPFC	sham	Migraine Disability Assessment Scale (MIDAS)	Sahu AK, et al 2019<br>*a double-blind sham-controlled study*		potentially better
**4**	Moisset, X.et al 2020	migraine	1Hz vertex rTMS (round coil)<br>1000 pulses, 1 session/d for 5 consecutive days	sham	reduction of migraine attacks compared with placebo	Teepker M , et al 2010<br>*sham controlled RCT, single blind, parallel groups*		no difference
**5**	Moisset, X.<br>Bouhassira, D.<br>et al<br>2020	poststroke pain	rTMS DLPFC active (F8c, parallel)<br>10 daily sessions (10Hz, 1250 pulses)	sham		de Oliveira RAA,et al 2014		no difference (from D1 to W4)
**5**	Moisset, X.<br>Bouhassira, D.<br>et al<br>2020	Central NP	rTMS, ACC, PSI or sham (double cone and H6 coils, parallel)<br>5 daily sessions than weekly for 11 weeks (10Hz, 1500pulses)	sham	NRS	Galhardoni R, et al 2019		no difference (at W12)
**6**	Liampas, A.et al 2020	intractable CPSP	rTMS	sham		de Oliveira,et al 2014		no difference
**7**	Gatzinsky, K. et al 2020	CPSP<br>*Spinal/brain stem lesion*	5 Hz rTMS	S1, PMC ,SMA,sham	VAS		Hirayama et al. 2006	potentially better

**8**	Aamir, A.<br>et al 2020	Brachial<br>Plexopathy	Site of stimulation：Peripheral: over the superior<br>trapezius muscle<br>Coil ：Double(70mm）<br>Protocol：10 daily sessions over 2 weeks. Two<br>protocols were applied (10 min apart)<br>For pain relief: stimulation at 15 Hz, with an intensity of<br>100% of the resting motor threshold was applied.Each<br>session had seven bursts. Each burst consisted of 150<br>pulses (10 s).<br>Interval between bursts was 20 s. In total, 1050 pulses<br>were delivered in each session<br>For strength increase: stimulation at 3 Hz, with an<br>intensity of 70% of the resting motor threshold was<br>applied. Each session had 50 bursts.<br>Each burst consisted of 30 pulses (10 s). Interval<br>between bursts was 30 s. In total, 1500 pulses were<br>delivered in each session	sham（active coil that<br>was elevated<br>away from the muscle,<br>rather than a sham coil<br>applied directly over the<br>muscle）	VAS	Khedr et al,2012	potentially better(at<br>least 1 month<br>after completion of<br>treatment)
**9**	Stilling, J. M.2019	chronic<br>migraine	I: 90% motor threshold (FDI) of dominant hand-2%<br>(≈equivalent to RMT)<br>f: 20Hz<br>#: 40 pulses of 10 trains with 30s ITI (400 total pulses)<br>-Figure 8 coil<br>-12 sessions on alternate days excluding weekends<br>Left DLPFC (5cm anterior to FDI motor hot spot)	sham	Attack<br>frequency,<br>headache index,<br>number of<br>abortive<br>medications	Brighina, F. et al,2004<br>*Parallel group, randomized,*<br>*double-blind, unreported*<br>*allocation.*	Potentially better
**9**	Stilling, J. M.2019	chronic<br>migraine	I: 110% RMT of FDI<br>f: 10Hz<br>#: 32 pulses with 30s ITI (1600 total pulses)<br>-Figure 8 coil<br>-Sham: coil perpendicular to vertex<br>-23 sessions over 8 weeks. Left DLPFC	sham	Pain intensity,<br>depression,<br>anxiety,<br>disability<br>(MIDAS)	Conforto AB., 2014<br>*Parallel group, randomized,*<br>*double-blind, sham-controlled,*<br>*concealed allocation, single-*<br>*center, proof-of-principle clinical*<br>*trial.*	Potentially<br>worse：<br>immediately；<br>Potentially<br>better：at 8 weeks
**9**	Stilling, J. M.2019	posttraumatic<br>headach	I: 80% RMT of ADM<br>f: 10 Hz<br>#: 20 trains with 1s ITI (2000 pulses)<br>-Figure 8 coil<br>-Sham: treatment side of the coil 180 degrees away<br>from the scalp after the RMT was determined<br>-Four rTMS sessions 24-72H apart Left DLPFC	sham	Intensity of<br>persistent daily<br>headache,<br>intensity and<br>duration of<br>debilitating	Leung, A. 2018<br>*Randomized, single blinded, sham*<br>*controlled, concealed allocation.*	Potentially<br>better：at 1 and 4<br>weeks
**9**	Stilling, J. M.2019	medication over	I: Visual motor threshold (FDI) of dominant hand-2%<br>(≈equivalent to RMT)<br>f: 1Hz<br>#: 500 pulses of 2 trains with 60s ITI (1000 total pulses)<br>-Round coil<br>-5 sessions on consecutive days. Vertex	sham	Reduction in<br>headache<br>attacks<br>(HA/8wks)	Teepker M., 2010<br>*Randomized, sham controlled,*<br>*single blinded, unconcealed*<br>*allocation.*	No difference

**9**	Stilling, J. M.2019	Headache	dTMS<br>I: 100% MT (APB)<br>f: 10Hz<br>#: 36 pulses of 10 trains with 20s ITI (360 total pulses)<br>-H1 coil<br>-3 sessions on alternate days, for a total of 12 sessions over 1 month<br>Control group: medically managed (preventative & abortive) Bilateral DLPFC (5.5cm anterior to APB hot spot)	sham	Headache frequency (mean over no reported duration)	Rapinesi, C. 2016		Potentially better: for 6 wks
**9**	Stilling, J. M.2019	Headache	sTMS -0.9T single magnetic field pulse<br>-2 pulses 30 s apart<br>-Rise time 180 µs<br>-Total pulse length ≤1 ms<br>-Treatment delivered ASAP after aura began and always within 1 h of aura onset for max 3 attacks.<br>-Sham: similar sound, no magnetic pulses<br>Occipital cortex	sham	Pain-free response 2 h after the first attack, non-inferiority at 2 h for nausea, photo/phono-phobia.	Lipton, R. 2010		Potentially better: after 2 h
**9**	Stilling, J. M.2019	Headache	sTMS - Single pulse 2.3 T, 187V peak induced voltage, pulse length 70µsec.<br>-2 pulses, 5 seconds apart<br>-Experimental: high stimulation at 50% max output<br>-Control: low stimulation at 30% max output<br>Applied over area of perceived pain or region of aura generation	sham	Pain intensity (0-5 Likert type scale), measuring and assessing suffering questionnaire (MASQ), heart rate variability	Clarke, B.M. 2006		No difference
**9**	Stilling, J. M.2019	Headache	sTMS - Single pulse 0.9 T, 180 µsec rise time, total pulse<br>length < 1 ms.<br>-Patients treated avg. 13 attacks/month Occiput	sham	Migraine days (head pain of moderate or severe intensity, pain scale >4/10, lasting at least 4 hours)		Bhola. R, 2015	Potentially better:at 12 wks
**9**	Stilling, J. M.2019	Headache	sTMS -0.9T single magnetic field pulse<br>-2 pulses 30 s apart<br>-Rise time 180 µs<br>-Total pulse length ≤1 ms<br>-Preventative Rx: 2 pulses delivered 2x/day<br>-Acute Rx: 3 pulses +/- 2 pulses if no resolution in 15 mins Occiput	sham	Mean reduction in headache days from baseline to statistically derived placebo estimate.		Starling, A. 2018 prospective cohort: non-randomized, non-blinded (open label), non-controlled trial.	Potentially better
**10**	Reuter, U.2019	acute migraine	sTMS	sham	NA	Lipton RB,2010		Potentially better
**10**	Reuter, U.2019	Migraine	sTMS	historical controls	NA		Starling et al,	Unclear

**11**	Ramger, B. C.et al 2019	Central Post-Stroke Pain	rTMS<br>Stimulation Location：Primary motor cortex<br>Intensity：120% RMT<br>Current Flow：10 Hz − 25 × 5 sec<br>Frequency of Intervention：1×/day<br>Length of Intervention：10 days	sham	VAS	De Oliveira et al, 2014<br>*Prospective cohort*		no difference
**12**	Hamid, P.et al 2019	chronic neuropathic pain	10HZ、DLPFC		VAS		Borckardt et al, 2009<br>*Cross-over*	potentially better（immediate and short-termed）
**12**	Hamid, P.et al 2019	visceral pain	1 HZ、Somatosensory cortex (SII)		VAS	Fregni et al ,2011<br>*Parallel RCT*		potentially better
**12**	Hamid, P.et al 2019	chronic widespread pain	10 HZ、left dorsolateral prefrontal (LDLPFC)			Avery et al, 2015<br>*Parallel RCT*		Unclear
**12**	Hamid, P.et al 2019	migraine	High frequency、DLPFC			Conforto et al,2014<br>*Parallel*		no difference
**13**	Feng, Y.et al 2019	Chronic migraine (IHS)	20 HZ,LDLPFC, 90% RMT LDLPFC	Coil rotation (vertical to LDLPFC)	Headache frequency Headache index	Brighina et al,2004		Potentially better
**13**	Feng, Y.et al 2019	Migraine	1 HZ，Vertex ,100% RMT Vertex	Sham coil	Headache frequency Pain intensity (0-10)	Teepker et al 24		No difference
**13**	Feng, Y.et al 2019	Chronic migraine	10 Hz,LDLPFC ,110% RMT LDLPFC	Coil rotation (vertical to v	Headache frequency Pain intensity (0-10)	Conforto et al,2014		No difference
**13**	Feng, Y.et al 2019	Chronic migraine	10 Hz, Bilateral DLPFC, 100% RMT dTMS	/	Headache frequency Pain intensity NRS (0-10)	Rapinesi et al,2016		Potentially better
**14**	O'Connell, N. E. et al 2018	chronic widespread pain	rTMS<br>Stimulation parameters: frequency 10 Hz; coil orientation not specified; 120% RMT;<br>number of trains 75; duration of trains 4 s; ITI 26 s; total number of pulses 3000<br>Stimulation location: L DLPFC<br>Number of treatments: 15 sessions over 4 weeks	sham	NRS	Avery et al, 2015		no difference
**14**	O'Connell, N. E. et al 2018	peripheral neuropathic pain	rTMS, figure-of-8 coil<br>Stimulation parameters: frequency 10 Hz; coil orientation not specified; 100% RMT;<br>number of trains 40; duration of trains 10 s; ITI 20 s; total number of pulses 4000<br>Stimulation location: L PFC<br>Number of treatments: 3 over a 5-d period	sham	average daily pain 0-10 Likert scale, anchors “no pain at all” to “worst pain imaginable”		Borckardt et al,2009	potentially better

**14**	O'Connell, N. E. et al 2018	CPSP	rTMS<br>Stimulation parameters: frequency 10 Hz; coil orientation not specified, 120% RMT,<br>number of trains 25; duration of trains 5 s; ITI 25s; total number of pulses 1250<br>Stimulation location: L premotor/DLPFC<br>Number of treatments: 10 sessions daily for 2 weeks	sham	NRS	de Oliveira et al,2014		no difference
**14**	O'Connell, N. E. et al 2018	chronic pancreatitis pain	rTMS, figure-of-8 coil<br>Stimulation parameters: frequency 1 Hz or 20 Hz; coil orientation not specified; 90%<br>RMT; number of trains not specified; duration of trains not specified; ITI not specified;<br>total number of pulses 1600<br>Stimulation location: L and R SII<br>Number of treatments: 1 for each condition	sham "specially designed sham coil".	VAS		Fregni et al,2005	Unclear
**14**	O'Connell, N. E. et al 2018	chronic pancreatitis pain	rTMS<br>Stimulation parameters:frequency 1 Hz; coil orientation not specified, number of trains<br>1; duration of trains not specified; intensity 70% maximum stimulator output, total<br>number of pulses 1600<br>Stimulation location: SII<br>Number of treatments: 10, x 1 daily (weekdays only)	sham	VAS	Fregni et al,2011		Unclear
**14**	O'Connell, N. E. et al 2018	intractable deafferentation pain (mixed central, peripheral and facial)<br>Prior management details: intractable	Stimulation type: rTMS, figure-of-8 coil<br>Stimulation parameters: frequency 5 Hz; coil orientation not specified; 90% RMT;<br>number of trains 10; duration of trains 10 s; ITI 50 s; total number of pulses 500<br>Stimulation location: condition 1: M1; condition 2: primary sensory cortex; condition<br>3: pre-motor area; condition 4: supplementary motor area; condition 5: sham<br>Number of treatments: 1 for each condition<br>Control type: coil angled 45º from scalp with synchronised electrical scalp stimulations<br>to mask sensation				Hirayama 2006<br>Cross-over RCT;	no difference
**14**	O'Connell, N. E. et al 2018	below level post SCI, predominantly neuropathic pain	rTMS<br>Stimulation parameters: frequency 10 Hz; coil orientation AP direction, 120% RMT ,<br>number of trains 25; duration of trains 5 s; ITI 25s; total number of pulses 1250<br>Stimulation location: L PFC (no neuronavigation)<br>Number of treatments: 10 sessions daily x 5 per week for 2 weeks	sham coil - same sound a	VAS	Nardone et al,2017		potentially better
**14**	O'Connell, N. E. et al 2018	burning mouth syndrome	rTMS<br>Stimulation parameters: frequency 10 Hz; coil orientation not specified, 100% RMT,<br>number of trains 10; duration of trains 5 s; ITI 10 s; total number of pulses 3000<br>Stimulation location: L DLPFC<br>Number of treatments: 10 x 1 daily on work days	sham coil - same sound and appearance and sensory cues	NRS	Umezaki,et al 2016		Unclear

**15**	Herrero Babiloni, A. et al.2018	Trigeminal neuropathy, atypical facial pain	rTMS<br>Target area :Left prefrontal cortex<br>Frequency, 10 Hz, 100% MEPs<br>Pulses/session, no. sessions: 4000/session, 3 sessions				Borckardt et al, 2009		potentially better
**15**	Herrero Babiloni, A. et al.2018	Trigeminal neuropathic pain, atypical facial pain, and BMS	rTMS<br>Target area :a) Contralateral S1/M1 representation face area if symptoms unilateral and right S1/M1 if bilateral b) S2/M2 right side<br>Frequency, 10 Hz, 90% MEPs<br>Pulses/session, no. sessions: 1000/session, 1 session				Lindholm et al, 2015		potentially better
**15**	Herrero Babiloni, A. et al.2018	BMS	rTMS<br>Target area :Left prefrontal cortex<br>Frequency, 10 Hz, 110% MEPs<br>Pulses/session, no. sessions: 3000/session, 10 sessions			Umezaki et al, 2016			potentially better(maintained 60 days)
**16**	Lan, L.2017	Migraine	sTMS 2 pulses about 30s occiput	sham			Richard et al.2010		Potentially better：migraine with aura
**16**	Lan, L.2017	Migraine	23 sessions of active rTMS-DLPFC, total of 1600 pulses per session	sham		Adriana et al. 2014			No difference
**16**	Lan, L.2017	Migraine	Tabletop clinic- based rTMS over left frontal cortex 10 Hz rTMS, 600 pulses in 10 trains	sham			Usha et al. 2013		Potentially better
**16**	Lan, L.2017	Migraine	Tabletop clinic- based rTMS-DLPFC 12 rTMS sessions, each rTMS session consisted of 10 trains of 2-s duration, separated by 30-s pause, given at 20-Hz frequency and 90% MT intensity	sham			Filippo et al. 2004		No difference
**17**	Kumru, H. et al.2017	CPSP, SCI, TGNI, PNI, RA	2 sessions/5Hz or sham/90% RMT/500pulses/M1,	PMC, SMA, S1,sham	VAS, SF-MPQ			Saitoh et al. 2006	potentially better (M1,maintained 3 hours)
**17**	Kumru, H. et al.2017	CPSP, SCI, TGNP, PNP	1 session/5 Hz or sham/90% RMT/500 pulses/S1, PMC or SMA	S1, PMC ,SMA,sham	VAS			Hirayama et al. 2006	potentiall better
**17**	Kumru, H. et al.2017	PNP	3 sessions/10 Hz or sham/100% RMT/4000 pulses/left DLPFC		NRS, QTPT, QMPT			Borckardt et al. 2009	potentiall better
**17**	Kumru, H. et al.2017	SCI, PL, CNP, PNP	15 sessions/1 Hz/110% RMT/1600 pulses/right DLPFC		VAS			Sampson et al. 2011	potentiall better(3 months)
**17**	Kumru, H. et al.2017	CPSP	10 sessions/10Hz or sham/120% RMT/1250 pulses/ PMC, DLPFC		VAS, MPQ			De Oliveira et al. 2014	no difference
**17**	Kumru, H. et al.2017	OFP	1 session/10Hz or sham/90% RMT/1000 pulses/ contralateral S1-M1 or right S2		NRS, BPI			Lindholm et al. 2015	potentiall better
**17**	Kumru, H. et al.2017	BPL	10 sessions/3Hz, 15 Hz or sham/100% RMT/1500 pulses/superior trapezius muscle		VAS			Khedr et al. 2012	potentiall better(1 month)

**18**	Goudra, B.2017	Neuropathic pain gastric bypass surgery	10HZ，1 sessions-4000/ session	Sham rTMS				Jeffrey J. Borckardt et al. 2006	No difference
**19**	Shirahige, L.et al 2016	Migraine	HF-rTMS (left DLPFC);20Hz，90% of resting motor threshold，5 cm anterior to the optimal site for right FDI；Duration of Sessions (Frequency):12 (3/week)					Brighina et al, 2004	Potentially better:after 1 month
**19**	Shirahige, L.et al 2016	Migraine	EG: HF-rTMS (left DLPFC) CG: HF-rTMS (vertex) 10Hz,110% of resting motor threshold,5 cm anterior to the optimal site for right FDI； Duration of Sessions (Frequency):12 (3/week)：23 (3/week)					Conforto et al, 2014	Potentially better:to week 8;No difference to week 4.
**19**	Shirahige, L.et al 2016	Migraine	s-TMS；0.9 T,Just below the occipital bone；Oz					Lipton et al, 2010	Potentially better:at 24h and 48h
**19**	Shirahige, L.et al 2016	Migraine	LF-rTMS(vertex)；1 HZ，98% of visual motor threshold,98% of visual motor threshold；Duration of Sessions (Frequency):12 (5 sessions/ week)	sham coil				Teepker et al, 2010	Potentially better：during 8 weeks
**20**	Cragg, J. J.et al 2016	Stroke and multiple sclerosis pharmaceutical	rTMS	placebo	VAS	de Oliveira et al.,2014			no difference
**21**	Chen, CC,et al 2016	central post-stroke pain (CPSP)	10 Hz TMS	sham	VAS	De Oliveira et al., 2014			Potentially better：in 1 to 10 days
**21**	Chen, CC,et al 2016	central post-stroke pain (CPSP)	5 Hz TMS	S1, PMC ,SMA,sham	NA			Hirayama et al., 2006	Potentially better
**22**	Jin, Y. .et al 2015	NP	Figure8/M1& 5Hz, 90% RMT, 50 pulses × 10 trains, ITI = 50s	S1, PMC ,SMA,sham	VAS		Hirayama 2006 *Cross-over sham control*		potentially better
**23**	Galhardoni, R. et al 2015	Postoperative (nZ20)	Type of Coil, Target, Stimulation Intensity, and Frequency (Total No. of Pulses):F8, left PFC, 100% RMT, 10Hz (4000p) Orientation of the Induced Current:NR No. of Sessions:1 Responders in the Active Group (% of Pain Intensity Reduction:NR Responders in the Control Group (% of Pain Intensity			Borckardt et al ,2006 *Double-blind,placebo controlled*			potentially better

**23**	Galhardoni, R. et al 2015	NP: central (nZ14); peripheral (nZ6)	Type of Coil, Target, Stimulation Intensity, and Frequency (Total No. of Pulses):F8; M1, S1, PMC, and SMA; 90% of RMT of painful area (or 100A/ ms); 5Hz and sham (500p)<br>Orientation of the Induced Current:NR<br>No. of Sessions:1<br>Responders in the Active Group (% of Pain Intensity Reduction:M1: 10/20 patients (>30% pain intensity reduction)<br>Responders in the Control Group (% of Pain Intensity Reduction:No effect for S1, SMA, and PMC	S1, PMC ,SMA,sham		Hirayama et al,2006<br>*Double-blind, controlled, crossover*	potentially better(up to 3h)
**23**	Galhardoni, R. et al 2015	Chronic visceral pain (pancreatitis) (nZ17)	Type of Coil, Target, Stimulation Intensity, and Frequency (Total No. of Pulses):F8, SII, 70% RMT, 1Hz (1600p)<br>Orientation of the Induced Current: NR<br>No. of Sessions: 10<br>Responders in the Active Group (% of Pain Intensity Reduction: 1Hz: NR (week 1: 27.2% pain intensity reduction)<br>Responders in the Control Group (% of Pain Intensity Reduction:Sham: NR (1.1% increase in pain intensity)		Fregni et al,2011<br>*Double-blind, sham controlled*		potentially better
**23**	Galhardoni, R. et al 2015	NP: spinal cord injury (nZ17)	Type of Coil, Target, Stimulation Intensity, and Frequency (Total No. of Pulses):F8, vertex, 110% RMT, 10Hz (1500p)<br>Orientation of the Induced Current:Tangentially to scalp over the vertex<br>No. of Sessions: 10<br>Responders in the Active Group (% of Pain Intensity Reduction:10Hz: NR (pain intensity reduction) 10d: 5.0/10; 6wk: 5.0/10; 6mo: 7.0/10<br>Responders in the Control Group (% of Pain Intensity Reduction: Sham: NR (pain intensity reduction 10d: 6.0/10 6wk: 7.0/10 6mo: 7.0/10)		Yılmaz et al 107 2014<br>*Double-blind, randomized, sham controlled*		no difference
**24**	Leung, A. et al 2009	1NR；1PN 8PSP；3SC 3TGN	500 Low 5 Hz Low 1 Single	S1, PMC ,SMA,sham		Hirayama et al,2006	potentially better (2week maintain)
